# Let them eat fruit! The effect of fruit and vegetable consumption on psychological well-being in young adults: A randomized controlled trial

**DOI:** 10.1371/journal.pone.0171206

**Published:** 2017-02-03

**Authors:** Tamlin S. Conner, Kate L. Brookie, Anitra C. Carr, Louise A. Mainvil, Margreet C. M. Vissers

**Affiliations:** 1 Department of Psychology, University of Otago, Dunedin, Otago, New Zealand; 2 Centre for Free Radical Research, Department of Pathology, University of Otago, Christchurch, New Zealand; 3 Department of Human Nutrition, University of Otago, Dunedin, Otago, New Zealand; TNO, NETHERLANDS

## Abstract

This study tested the psychological benefits of a 14-day preregistered clinical intervention to increase fruit and vegetable (FV) consumption in 171 low-FV-consuming young adults (67% female, aged 18–25). Participants were randomly assigned into a diet-as-usual control condition, an ecological momentary intervention (EMI) condition involving text message reminders to increase their FV consumption plus a voucher to purchase FV, or a fruit and vegetable intervention (FVI) condition in which participants were given two additional daily servings of fresh FV to consume on top of their normal diet. Self-report outcome measures were depressive symptoms and anxiety measured pre- and post-intervention, and daily negative and positive mood, vitality, flourishing, and flourishing behaviors (curiosity, creativity, motivation) assessed nightly using a smartphone survey. Vitamin C and carotenoids were measured from blood samples pre- and post-intervention, and psychological expectancies about the benefits of FV were measured post-intervention to test as mediators of psychological change. Only participants in the FVI condition showed improvements to their psychological well-being with increases in vitality, flourishing, and motivation across the 14-days relative to the other groups. No changes were found for depressive symptoms, anxiety, or mood. Intervention benefits were not mediated by vitamin C, carotenoids, or psychological expectancies. We conclude that providing young adults with high-quality FV, rather than reminding them to eat more FV (with a voucher to purchase FV), resulted in significant short-term improvements to their psychological well-being. These results provide initial proof-of-concept that giving young adults fresh fruit and vegetables to eat can have psychological benefits even over a brief period of time.

**Trial registration:** Australian New Zealand Clinical Trials Registry ACTRN12615000183583

## Introduction

The physical health benefits of fruit and vegetables are well established. People who eat more fruit and vegetables (FV) have better cardiovascular health [1], reduced risk of some cancers [2], and greater longevity than people who eat fewer FV [3]. There is also growing evidence that people who eat more FV have better mental health. Higher consumption of FV is correlated with several psychological outcomes including a lower incidence of depression and anxiety [4, 5, 6, 7, 8, 9], greater happiness [10, 11], higher life satisfaction [10, 12, 13, 14], and greater social-emotional well-being or “flourishing” [15]. This growing body of research is intriguing because it suggests that the foods people eat have a much broader impact beyond the notable physical health benefits. However, studies examining the psychological benefits of FV have been predominantly observational and epidemiological to date, with a relative lack of intervention research that would support a causal link.

Prospective research designs provide some evidence that increasing FV consumption may cause psychological benefits. At least five longitudinal studies found that dietary improvements including a diet higher in FV (i.e., a Mediterranean diet) predicted subsequent reductions in depression among adolescents, adults, and older adults [16, 17, 18, 19, 20]. However, these studies have not typically separated the effects of FV from other components of the Mediterranean diet such as olive oil or fish. More relevant are the results from Mujcic and Oswald [14] who analysed the prospective relationship between FV consumption and happiness and life satisfaction from the 2007 and 2009 waves of the Household, Income, and Labour Dynamics in Australia (HILDA) study, a nationally representative panel survey of over 12,000 people aged 15 years and older. They found that that increased FV consumption predicted greater “happiness gains” and increases in life satisfaction over the two years. Respondents with the largest increases in FV reported the greatest gains in well-being over time [14]. The opposite direction–happiness or life satisfaction predicting subsequent increases in FV consumption–was not found.

Only a few intervention studies have tested the psychological benefits of increased FV consumption directly. One study of 271 low-income participants in the UK showed improvements in the composite measure of mental health from the Rand Short Form Survey (SF-36 [21]) from baseline to 8-weeks follow up in participants who underwent brief nutritional and behavioral counselling to increase their FV consumption [22]. Another study of 35 healthy young men showed that eating two kiwifruits every day for six weeks resulted in modest improvements to mood as measured by the Profile of Mood States (POMS) questionnaire [6, 23]. Mood improvements corresponded with increased vitamin C levels, were only significant for the young men with poorer mood at the start of the study, and were mainly driven by reductions in fatigue, increases in “vigor” (which included reports of feeling cheerful, energetic, lively, and full of pep), and decreases in depressive symptoms (trend only) [6]. A third study of 100 university students showed that snacking on one piece of fruit (either an apple, large clementine, or banana) each day for 10 days resulted in reductions in fatigue and anxiety compared to daily snacking on chocolate wafers or potato chips [24]. Taken together, these studies support a causal link between FV and various measures of mental health and mood. Yet each study is limited in some way–either by a small sample size [6], exclusive focus on fruit [6, 24], or use of a composite mental health index that does not separate aspects of mental health such as depression and anxiety [22]. And, to our knowledge, no intervention study has tested whether FV improves aspects of well-being aside from the impact on ill-being. It is important to fill this gap given the growing observational and prospective evidence linking FV to indicators of well-being such as happiness and life satisfaction [10, 12, 13, 14], optimism [25], and flourishing [15].

Accordingly, in the present study, we tested the psychological benefits of a 14-day preregistered clinical intervention designed to increase fruit and vegetable (FV) consumption among low-FV-consuming young adults [26]. We defined low-consuming young adults as anyone aged 18 to 25 who reported eating fewer than 3 combined servings of fruit and vegetables per day. This cut-off reflects lower intake than the minimum national dietary guidelines of 5 combined servings of FV per day [27]. We implemented our intervention on a young adult population because young adults typically have the lowest FV consumption of all the age groups [28, 29, 30] and they are developing early autonomy over their health behaviors [31]. More details on the development of the intervention can be found in Brookie et al. [26]. To briefly summarize, we ran a three-arm randomized clinical trial (RCT) (n = 171) with three conditions: a diet-as-usual control condition in which participants were asked to maintain their regular food consumption for two weeks; an ecological momentary intervention condition (EMI), in which participants were sent twice daily text-messages that utilized a variety of behavioral change techniques to help them increase their fruit and vegetable consumption to at least 5 combined servings a day (plus they were given a voucher to purchase the FV); or a fruit and vegetable condition intervention (FVI), in which participants were given a bag of two weeks’ worth of fruit and vegetables (kiwifruit or oranges depending on the season, apples, and carrots) and were asked to consume at least two additional servings (1 fruit and 1 vegetable) on top of their regular daily FV consumption. Participants in both intervention conditions reported significantly higher daily FV consumption (3.7 servings / day) compared to control participants (2.8 servings / day), and compared to their own baseline (2.5 servings / day) when FV was reported nightly for two weeks using a smartphone-accessed survey [26]. Moreover, blood samples taken before and after the intervention showed that participants in the two experimental groups had small gains in vitamin C levels and plasma carotenoids, suggesting that the self-reported changes in FV consumption were legitimate. Vitamin C and carotenoids are the most consistently reliable biomarkers of FV consumption and have been used to reflect intake of nutrient-dense FV [32].

The current paper presents the psychological outcomes of that same two-week intervention reported in Brookie et al. [26]. The primary outcome measures were depressive symptoms and anxiety measured pre- and post-intervention, as well as negative and positive mood, vitality, and flourishing measured nightly through a smartphone survey. Our choice of methodology—smartphone tracking of daily psychological outcomes for two weeks—reflected our desire to minimise memory-based reporting and maximise sensitivity to detect possible group differences in mood and well-being changes over time [33]. Our choice of outcome measures reflected a desire to measure both the negative and positive aspects of mental health [34]. We predicted that participants in the two experimental conditions (EMI and FVI) would show significant improvements in the psychological outcomes relative to participants in the control condition, and that the benefits of FV consumption would be stronger for the measures of well-being (positive mood, vitality, flourishing). Although previous research has linked FV to lower levels ill-being–such as reduced depression [9, 35] and anxiety [9, 24]–there is more evidence linking FV to higher levels of well-being such as positive mood, happiness, and flourishing [6, 10, 11, 12, 13, 14, 15]. We also included three unregistered “wild-card” variables in the daily smartphone survey–self-reported curiosity, creativity, and motivation–which we grouped under the umbrella term of flourishing behaviors. Higher FV consumption was previously shown to correlate with greater curiosity and creativity in young adults [15], but research has not yet established whether that relationship is causal. And, no research has tested whether FV might improve perceptions of motivation, a key correlate of vitality [36]. Lastly, we tested whether changes in the psychological outcomes would be mediated by two key biomarkers (vitamin C and carotenoids) and/or psychological expectancies. The mechanisms linking FV to well-being are unknown, but they may be due to key micronutrients like vitamin C and carotenoids, which act as cofactors for dopamine and other neurotransmitters involved in positive motivational states [37], or possibly due to positive psychological expectancies such as the perception that eating FV is virtuous or will make you feel better [38].

## Materials and methods

### Participants and procedure

**Fig 1:**
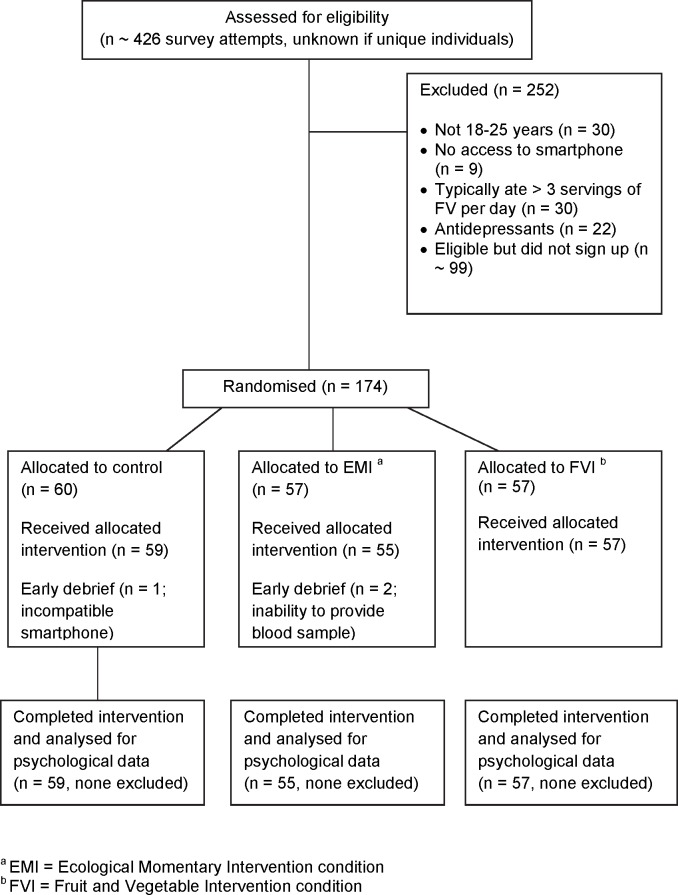
Participant Flow Diagram Depicting Reasons for Exclusion and Allocation to Intervention Conditions.

This is the same sample reported in Brookie et al. [26]. The experimental design and primary outcome measures were registered before recruitment with the Australia New Zealand Clinical Trials Registry (ANZCTRN12615000183583). Recruitment began in March 2015 and follow-up data was collected by the end of November 2015. Fig 1 shows the flow of participants throughout the trial and explanations for drop outs. Participants were 171 young adults (56 men; 115 women), 18 to 25 years old (*M* = 19.43, *SD* = 1.45) who identified as European (64%), Asian (18%), Māori or Pacific Islander (8%), or another or mixed ethnicity (11%). Participants were students at the University of Otago, New Zealand who were recruited through the Psychology Department’s experimental participation program and reimbursed with partial course credit (*N* = 135, 79%) or recruited through a student employment agency and reimbursed with a small cash payment (*N* = 36, 21%). Inclusion criteria included being in the young adult age range (18–25 years), having an Internet capable smartphone, identifying as low-FV-consuming (no more than 3 combined servings of FV per day), not being on any anti-depressant medication, and having no known FV allergies. All participants provided written consent to take part by reading and signing their name on a paper consent form (later kept in secured storage). The study and consenting procedures were approved by the University of Otago Human Ethics Committee (#15/010).

More detail about the intervention, including the development and complete list of intervention text-messages for the EMI condition, is described in Brookie et al. [26]. Participants attended an initial laboratory session and completed a computerized baseline survey that measured demographics and symptoms of depression and anxiety, among other measures. That evening, participants completed their first of 14 daily diary surveys delivered by hyperlink to their smartphones. The survey measured their daily mood and well-being (see *Measures*) and was accessible between 7:00 pm and 1:00 am. The next morning, participants attended a clinic in the Human Nutrition department where they were measured for height and weight using standardised techniques [39] and gave a fasting baseline blood sample (4 ml). Immediately after the clinic visit, participants randomly assigned to the EMI condition began to receive their twice-daily intervention messages to increase FV over the next 13 days. Participants in the FVI condition received their bag of FV to consume for the next 13 days. Participants in the control condition were asked to maintain their normal diet. After the 13 intervention days, participants returned to the Human Nutrition clinic and gave a follow up blood sample (4 ml), and completed follow-up measures of depressive symptoms and anxiety.

### Measures

Preregistered psychological outcome measures were depressive symptoms, anxiety, negative mood, positive mood, vitality, and flourishing. Curiosity, creativity, and motivation were not registered ahead of time and were therefore considered exploratory.

#### Demographics and covariates

Demographic measures were age, gender, and ethnicity. Height and weight measured during the first clinic visit was used to calculate body mass index (BMI) as a possible covariate. Smoking status was tested as a covariate in the vitamin C analysis because smoking increases the metabolism of vitamin C [40]. In the follow-up survey, participants were asked “How often do you currently smoke” by choosing one of five response options: *I don’t smoke now*, *less than once a month*, *at least once a month*, *at least once a week*, or *at least once a day*. Participants who smoked at least once a week or day were coded as smokers (1); all others were coded as non-smokers (0).

#### Depressive symptoms

Depressive symptoms were measured before and after the intervention using the 20-item Centre for Epidemiological Studies Depression Scale (CES-D; [41]). Participants rated their depressive symptomology “in the past week” on a 4-point scale ranging from 0 (*Rarely or none of the time*, *< 1 day*) to 3 (*Most or all of the time*, *5–7 days*). Responses were summed to give a total depressive symptom score ranging from 0 to 60 (pre α = 0.89; post α = 0.90).

#### Anxiety

Anxiety symptoms were measured before and after the intervention using the 7-item Anxiety sub-scale of the Hospital Anxiety and Depression Scale (HADS; [42]). Participants rated each item on how they “felt recently, including today” on a 4-point scale ranging from 0 (*Not at all* or *Only occasionally)* to 3 (*Very often* or *Most of the time*). Responses were summed to give a total anxiety score ranging from 0 to 21 (baseline α = 0.77; follow up α = 0.78).

#### Negative and positive mood

The daily survey included 3-item measures of negative mood (*sad*, *anxious*, *angry*) and positive mood (*relaxed*, *happy*, *enthusiastic*), which captured a range of activation states [43]. Participants rated each adjective on how they “felt today” on a 5-point Likert scale from 0 (*Not at all*) to 4 (*Extremely*). Responses were averaged across the three items each day for a measure of daily negative mood (α = 0.34) and daily positive mood (α = 0.42). The within-person reliabilities for the daily survey data were computed using procedures for nested data recommended by Nezlek [44]. Because of low reliability, we also analysed the items separately in supplementary analyses.

#### Vitality

The daily survey included a modified version of the 4-item energy/fatigue scale of the Rand 36-Item Short Form Survey (SF-36 [21, 45]). Participants were asked “today”: *Did you feel full of life? Did you have a lot of energy? Did you feel worn out? Did you feel tired?* We changed the first item from the original “full of pep” to “full of life” to better capture feelings of vitality and to connect with a young-adult population. Each item was rated on a 6-point scale labelled *None of the time*, *A little of the time*, *Some of the time*, *A good bit of the time*, *Most of the time*, and *All of the time*. Responses were coded from 0 to 100 in 20-point intervals following SF-36 guidelines (reverse-scoring *worn out* and *tired*), and summed within each day so that highest total score was 100, indicating higher vitality that day. The modified scale showed adequate within-person reliability (α = 0.70).

#### Flourishing

The daily survey included a shortened 3-item version of the 8-item Flourishing Scale that assesses feelings of engagement, purpose in life, and social-emotional connectedness, which previous research shows loads on a single “flourishing” factor [46]. We selected the three highest loading items from a previous dataset and adapted the items for a daily format. The three items were: *Today*, *I was engaged and interested in my daily activities*. *Today*, *I led a purposeful and meaningful life*. *Today*, *I was a good person and lived a good life*. Participants rated each item on a 7-point Likert scale from 1 (*Strongly disagree*) to 4 (*Neither agree nor disagree*) to 7 (*Strongly agree*). Responses were summed each day for a measure of daily flourishing ranging from 1 to 21 (α = 0.70).

#### Flourishing behaviors

The daily survey included three items answered on a 5-point Likert scale from 0 (*Not at all*) to 4 (*Extremely*). The first item measured curiosity: *How curious were you today? Did you seek out new things or experiences; look for opportunities to challenge yourself and grow as a person; or embrace the unfamiliar*. This question was guided by the content of the Curiosity and Exploration Inventory II [47]. The second item measured creative activity: *How creative were you today? Did you come up with novel or original ideas; express yourself in an original and useful way; or spend time doing artistic activities like*, *music*, *painting*, *writing*, *etc*. This question was based on scientific definitions of creativity [48] and has been used in previous research [15]. The third item measured perceived motivation: *How motivated were you today? Did you work towards your goals*, *or feel driven today?* This question was designed to capture a broad, conscious sense of motivation including working towards goals [49]. Responses to the three items were averaged each day for an index of daily flourishing behaviors (α = 0.28). Because of low reliability, these items were also analysed separately in supplementary analyses.

#### Vitamin C and carotenoids

A 4 ml fasting blood sample was taken before and after the intervention. Samples were kept in dark conditions on ice and centrifuged within 1 hour of collection. Plasma aliquots were stored at -80C until analysis. Plasma vitamin C was assessed by high-performance liquid chromatography (HPLC) with electrochemical detection, treated with reducing agent to recover total vitamin C. Total plasma carotenoids were assessed spectrophotometrically (for full blood processing details see [26]). Four people were unable to provide a post-intervention blood sample; therefore, analyses with vitamin C and carotenoids reflected a sample size of 167.

#### Psychological expectancies

The follow-up survey included questions about participants’ beliefs about the benefits and virtues of eating FV, as well as candy and fried foods as distractor items, and how participants thought eating these foods made them feel over the last two weeks. For analysis, we focused on the three items related to FV consumption: *Do you feel that eating fruits and vegetables is virtuous (righteous*, *good*, *moral)? Do you feel better about yourself when you eat more fruits and vegetables? Did you feel that eating fruits and vegetables improved your mood during these two weeks?* Each question was answered on a 5-point Likert scale from 0 (*Very slightly/not at all*) to 4 (*Extremely*). We added these expectancy items one third of the way through data collection; therefore, analyses with these items reflected a sample size of 121.

## Analyses and results

Table 1 presents the descriptive statistics for the demographic and outcome variables. The depression and anxiety scores were within norms for this population [50]. Compliance with the smartphone surveys was high at 90% (12.6/14 surveys completed; range 9 to 14 surveys). There were no differences between the control, EMI, and FVI conditions on survey compliance (*F*(2,168) = 0.91, *p* = 0.405), gender (χ^2^(2, *N* = 171) = 0.129, *p* = .938), age (*F*(2,168) = 1.24, *p* = 0.291), or BMI (*F*(2,168) = 1.18, *p* = 0.309). However, the conditions varied in ethnic composition (χ^2^(2, *N* = 171) = 8.31, *p* = .016) with more non-Europeans in the control condition (51%) than the two intervention conditions (EMI 29%; FVI 28%). Therefore, ethnicity was included as a covariate in the analyses. The analyses consisted of two Analyses of Covariance (ANCOVA) for the measures of depressive symptoms and anxiety, and six growth curve models for the daily measures of positive mood, negative mood, vitality, flourishing, and flourishing behaviors. Based on these seven main analyses, we applied a Bonferroni correction to the alpha level (.05/7 tests) to account for multiple hypothesis testing (*p* = .007).

**Table 1:** Sample Characteristics and Descriptive Statistics for the Psychological Outcome Variables.

	*M*	*SD*	Min	Max
*N*	171			
% Female	67.3			
% European	63.7			
Age	19.43	1.45	18	25
BMI	24.13	3.89	15.62	39.65
% Regular smoker ^a^	7.0			
Depressive symptoms (pre)	14.30	8.68	1.00	53.00
Depressive symptoms (post)	13.03	8.73	1.00	39.00
Anxiety (pre)	5.78	3.46	0.00	17.00
Anxiety (post)	5.37	3.31	0.00	17.00
Negative mood	0.77	0.46	0.00	2.18
Positive mood	2.05	0.50	0.57	3.92
Vitality	55.78	12.54	21.07	97.69
Flourishing	14.43	2.73	7.17	21.00
Flourishing behaviors ^b^	1.48	0.54	0.19	3.42
Curiosity	1.40	0.66	0.00	3.69
Creativity	1.13	0.67	0.00	3.82
Motivation	1.91	0.58	0.50	3.62

*Note*. SD = standard deviation; BMI = body mass index. Descriptive statistics for the daily variables of negative mood to motivation were computed on aggregated variables.

^a^ Regular smoker was defined someone who smokes at least once a week (n = 8) or once a day (n = 4).

^b^ Daily composite of curiosity, creativity, and motivation items.

Table 2 presents the raw scores for depressive symptoms and anxiety for participants in each condition. There were no differences at baseline between the three groups (depressive symptoms, *F*(2,168) = 0.19, *p* = 0.830; anxiety, *F*(2,168) = 0.88, p = 0.419). Results of a 3 (x 2) mixed ANCOVA with condition as the between-subjects variable, time as the within-subjects variable, and ethnicity as the covariate showed only a main effect of time in predicting depressive symptoms (*F*(1, 167) = 5.01, *p* = .027, Partial η^2^ = .029), no main effect of condition (*F*(2, 167) = 0.212, *p* = .809, Partial η^2^ = .003) and no time x condition interaction (*F*(2, 167) = 0.412, *p* = .663, Partial η^2^ = .005). Similar patterns were found for anxiety (time, *F*(1, 167) = 3.49, *p* = .063, Partial η^2^ = .020; condition, *F*(2, 167) = 1.33, *p* = .268, Partial η^2^ = .016; time x condition, *F*(2, 167) = 0.15, *p* = .859, Partial η^2^ = .002). The non-significant time x condition coefficients indicated that the interventions did not reduce depressive symptoms or anxiety relative to control.

**Table 2:** Changes in Depressive Symptoms and Anxiety for the Three Intervention Conditions, Unadjusted for Ethnicity.

		Baseline	Follow-up		
	*n*	*M* (*SD*)	*M* (*SD*)	Diff	95% *CI* diff
**Depressive Symptoms** ^**a**^					
Control	59	14.44 (9.82)	12.78 (10.07)	1.66	(0.09, 3.24)
EMI	55	13.73 (9.01)	13.07 (8.37)	0.66	(-1.57, 2.87)
FVI	57	14.70 (7.06)	13.25 (7.65)	1.46	(0.06, 2.85)
**Anxiety** ^**b**^					
Control	59	5.68 (3.66)	5.44 (3.54)	0.24	(-0.35, 0.83)
EMI	55	5.40 (3.17)	4.80 (3.02)	0.60	(-0.27, 1.47)
FVI	57	6.25 (3.50)	5.84 (3.29)	0.40	(-0.42, 1.23)

*Note*. *M* = mean; *SD* = standard deviation; EMI = ecological momentary intervention condition; FVI = fruit and vegetable intervention condition. Diff = point difference between baseline and follow-up group means. CI = Confidence Interval in the point difference between baseline and follow-up group means.

* *p* < 0.05

^a^ Depressive symptoms measured with the Center for Epidemiological Studies Depression Scale (CES-D).

^b^ Anxiety measured with the Hospital Anxiety and Depression Scale (HADS).

The daily survey data were analyzed using the Hierarchical Linear Modeling program to account for dependency among nested data (HLM v.6.08; [51]). We modelled linear growth curves to assess whether participants in the experimental conditions reported greater improvements in mood and well-being over time relative to participants in the control condition. Growth curves were modelled for each participant for each outcome using robust standard errors, as follows:

$$\begin{array}{l} Level\;1:\;Negative\;mood=B0+B1\;Time+r\\ Level\;2:\;B0=G00+G01\;ConditionD1+G02\;ConditionD2+G03\;Eth+u0\\ \;B1=G10+G11\;ConditionD1+G12\;ConditionD2+u1 \end{array}$$

This set of equations determined each person’s negative mood at baseline (B0), how negative mood changed over time for each participant (B1), and how these changes varied by experimental condition (G11; G12). Time was recoded so that 0 was day 1. G11 tested change in the EMI condition against change in the control condition (using Condition Dummy 1; control = 0; EMI = 1; FVI = 0). A significant G11 indicated that participants in the EMI condition exhibited a different pattern of change compared to participants in the control condition. G12 tested change in the FVI condition against the change in the control condition (using Condition Dummy 2; control = 0; EMI = 0; FVI = 1). A significant G12 indicated that participants in the FVI condition exhibited a different pattern of change compared to participants in the control condition. Thus, the null hypothesis was that participants in the EMI / FVI conditions would report similar changes over time as the control participants. For significant group differences, we plotted the growth patterns for the three groups and computed tests of the simple slopes. We also tested an additional set of dummy codes to compare the EMI and FVI groups in subsequent analyses. Ethnicity (0 = European; 1 = non-European) was entered as a covariate in the Level-2 intercept equation. Ethnicity was also entered as a covariate in the Level-2 slope equation but was not significant, did not affect results, and was removed from final models for simplicity.

Table 3 presents the growth curve results. Participants in the two experimental conditions did not show any improvements in negative or positive mood relative to participants in the control condition. Supplementary analyses showed similar null effects when analysing the mood items separately (see S2 Table) with an exception: *happy* showed significant group differences in change over time. Analysis of simple slopes showed that happiness was stable over time for participants in the FVI condition (*B*(*SE*) = -0.001(0.006), *p* = .845) but happiness decreased for participants in the EMI condition (*B*(*SE*) = -0.034(0.010), *p* = 0.001) and control condition (*B*(*SE*) = -0.012(0.008), *p* = 0.018). The slopes were significantly different only between the FVI and EMI conditions (*p* = 0.008).

**Table 3:** Results from Growth Curve Modelling Testing for Differences in Change over Time between the Three Intervention Conditions. Significant intervention effects are bolded (*p* < .007).

Outcome								
Negative Mood	G	Coef	*SE*	*p*		Coef	*SE*	*p*
Control Day 1	G00	0.744	0.068	< 0.001				
EMI Day 1 diff	G01	-0.068	0.095	0.471				
FVI Day 1 diff	G02	0.049	0.093	0.601				
Ethnicity	G03	-0.024	0.079	0.758				
Control Change (Δ) ^a^	G10	0.011	0.006	0.085				
EMI Change diff ^b^	G11	-0.001	0.010	0.897	EMI Δ ^d^	0.009	0.007	0.204
FVI Change diff ^c^	G12	-0.012	0.008	0.136	FVI Δ diff ^e^	-0.011	0.009	0.897
Positive Mood	G	Coef	*SE*	*p*				
Control Day 1	G00	2.137	0.092	< 0.001				
EMI Day 1 diff	G01	0.194	0.122	0.115				
FVI Day 1 diff	G02	0.006	0.103	0.955				
Ethnicity	G03	-0.140	0.084	0.098				
Control Change	G10	-0.014	0.008	0.072				
EMI Change diff	G11	-0.013	0.012	0.261	EMI Δ	-0.027	0.008	0.001
FVI Change diff	G12	0.006	0.009	0.529	FVI Δ diff	0.019	0.011	0.076
Vitality	G	Coef	*SE*	*p*				
Control Day 1	G00	58.639	2.443	< 0.001				
EMI Day 1 diff	G01	-0.701	2.915	0.810				
FVI Day 1 diff	G02	-6.778	2.976	0.024				
Ethnicity	G03	-0.510	2.140	0.812				
Control Change	G10	-0.336	0.204	0.101				
EMI Change diff	G11	0.144	0.277	0.603	EMI Δ	-0.192	0.187	0.307
** FVI Change diff**	**G12**	**0.791**	**0.284**	**0.006**	FVI Δ diff	0.647	0.027	0.019
Flourishing	G	Coef	*SE*	*p*				
Control Day 1	G00	14.505	0.509	< 0.001				
EMI Day 1 diff	G01	0.440	0.588	0.455				
FVI Day 1 diff	G02	-0.630	0.611	0.305				
Ethnicity	G03	-0.200	0.470	0.671				
Control Change	G10	-0.031	0.027	0.242				
EMI Change diff	G11	-0.007	0.041	0.864	EMI Δ	-0.038	0.031	0.205
** FVI Change diff**	**G12**	**0.128**	**0.042**	**0.003**	**FVI Δ diff**	**0.135**	**0.045**	**0.004**
Flourishing Behaviors	G	Coef	*SE*	*p*				
Control Day 1	G00	1.548	0.100	< 0.001				
EMI Day 1 diff	G01	-0.079	0.111	0.476				
FVI Day 1 diff	G02	-0.110	0.117	0.350				
Ethnicity	G03	0.044	0.096	0.647				
Control Change	G10	-0.012	0.006	0.038				
EMI Change diff	G11	0.003	0.008	0.756	EMI Δ	-0.009	0.006	0.093
** FVI Change diff**	**G12**	**0.025**	**0.009**	**0.006**	FVI Δ diff	0.022	0.009	0.011

*Note*. Coef = coefficient from Hierarchical Linear Modeling; SE = Robust standard error; diff = difference in coefficient; EMI = ecological momentary intervention condition; FVI = fruit and vegetable intervention condition; Δ = change. Degrees of freedom were 167 for G00 –G03 and 168 for G10 –G12.

^a^ Change over time for reference group (control condition).

^b^ Difference in change between EMI condition versus control condition.

^c^ Difference in change between FVI condition versus control condition.

^d^ Change over time for reference group (EMI condition).

^e^ Difference in change between FVI condition versus EMI condition.

**Fig 2:**
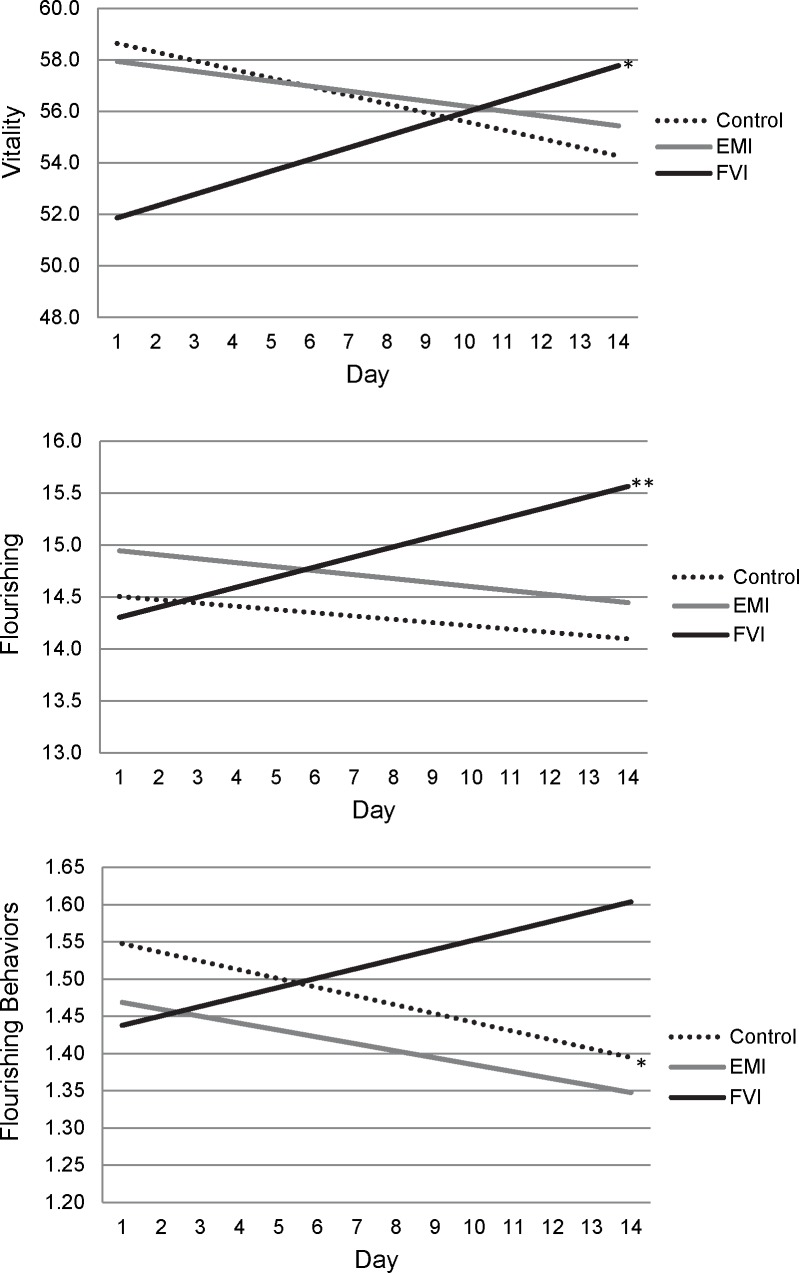
Changes in vitality, flourishing, and flourishing behaviors (composite of daily curiosity, creativity, and motivation) for the control, ecological momentary intervention (EMI), and fruit and vegetable intervention (FVI) conditions. Significant simple slopes are indicated. * *p* < .05; * *p* < .01.

**Fig 3:**
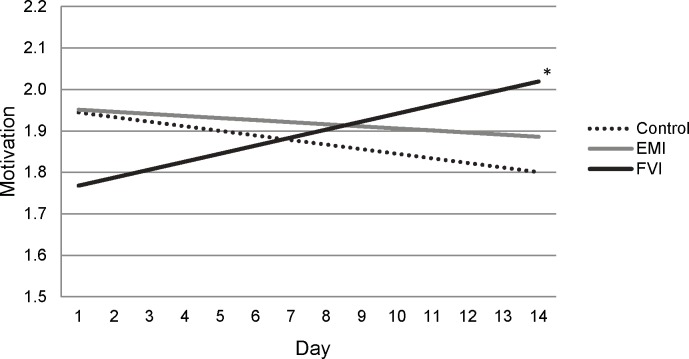
Changes in self-reported motivation for the control, ecological momentary intervention (EMI), and fruit and vegetable intervention (FVI) conditions. Significant simple slopes are indicated. * *p* < .05.

However, participants in the FVI condition did show improvements to their psychological well-being compared to participants in the EMI and control conditions. As shown in Table 3, participants given fresh fruit and vegetables to consume (FVI condition) reported significant growth in vitality, flourishing, and flourishing behaviors compared to participants in the EMI and control conditions. Fig 2 presents the growth patterns for these outcomes. As shown in the top of Fig 2, participants in the FVI condition reported lower starting vitality than the other groups, but their rate of improvement was greater compared to both the EMI and control conditions. Analysis of simple slopes showed that participants in the FVI condition reported significant growth in vitality over time (*B*(*SE*) = 0.455(0.200), *p* = .023) whereas participants in EMI and control conditions were unchanged over time (EMI *B*(*SE*) = -0.192(0.187), *p* = 0.307; control *B*(*SE*) = -0.336(0.204), *p* = 0.101). Group differences were even more apparent for the measure of flourishing. As shown in the middle of Fig 2, participants in the FVI condition reported significantly greater increases in flourishing compared to participants in the control and EMI conditions. Analysis of the simple slopes showed that participants in the FVI condition reported significant growth in flourishing over time (*B*(*SE*) = 0.010(0.033), *p* = .004) whereas participants in the EMI and control conditions were unchanged over time (EMI *B*(*SE*) = -0.038(0.031), *p* = 0.215; control *B*(*SE*) = -0.031(0.027), *p* = 0.242). And, shown in the bottom of Fig 2, participants in the FVI condition also reported significantly greater growth in flourishing behaviors compared to participants in the control condition. Simple slope analyses revealed a trend growth in flourishing behaviors for participants in the FVI condition (*B*(*SE*) = 0.013(0.007), *p* = .053), a trend decrease for participants in the EMI condition (*B*(*SE*) = -0.009(0.006), *p* = .093), and a significant decrease for participants in the control condition (*B*(*SE*) = -0.012(0.006), *p* = .038). When we analyzed the three flourishing behaviors separately, all three showed similar patterns, but the strongest effects were found for motivation (see *S2*). Fig 3 presents the growth patterns for motivation showing that participants in the FVI condition reported significantly increasing motivation over time (*B*(*SE*) = 0.019(0.010), *p* = .045), whereas participants in the EMI and control conditions were unchanged over time (EMI *B*(*SE*) = -0.005(0.011), *p* = 0.652; control *B*(*SE*) = -0.011(0.008), *p* = 0.218).

In terms of effect sizes, being assigned to the FVI condition (vs. control) predicted 8.7% of the variance in growth in vitality, 10.5% of the growth in flourishing, and 18.4% of the growth in flourishing behaviors using variance-explained effect size estimates of the time slopes [52].

Additional analyses testing for non-linear change over time using time and time squared as level-1 predictors found no evidence for curvilinear changes over time or moderation of curvilinear changes by intervention group. Thus, changes in well-being as a function of the intervention were strictly linear.

Lastly, none of these changes in well-being were mediated by post-test vitamin C, post-test carotenoid levels, or psychological expectancies. Following procedures for testing mediation with a multicategorical independent variable [53], we added each mediator as a separate Level-2 predictor (grand-mean centered) plus the two condition dummy codes (ConditionD1 and ConditionD2) to predict both the intercepts (B0s) and time slopes (B1s) for vitality, flourishing, and flourishing behaviors (with ethnicity as a covariate in the intercept equation, as before). Five mediators were tested separately: post-test vitamin C levels, post-test carotenoid levels, expectancy 1 (eating FV is virtuous), expectancy 2 (I feel better about myself when eating more FV), and expectancy 3 (eating FV improved my mood). None of the condition coefficients G11 or G12 changed when entering vitamin C, carotenoids, or the three psychological expectancy questions into the model. The fact that the total effects (c) were nearly equivalent to the direct effects (c’) when partialling out each indirect mediational pathway (ab) argues against mediation by these variables.

## Discussion

Giving young adults fruits and vegetables (FV) to eat, but not reminding them to eat their FV, improved several aspects of psychological well-being over a two-week period. Despite both intervention groups reporting modestly higher and similar consumption of FV relative to control (3.7 vs. 2.8 daily servings) [26], only young adults who were given two weeks’ worth of FV showed improvements in their feelings of vitality, flourishing, and motivation. The short duration of our study indicated that FV intake translated into improved well-being quite rapidly.

The intervention effects were prominent across measures of well-being but not ill-being such as depression, anxiety, or negative mood. This is an interesting pattern that supports the observational and prospective research linking FV to greater well-being [10, 14, 15]. It is also possible that this pattern might reflect differences in the timescale of effects. The majority of research linking depression to dietary patterns has been longitudinal, meaning that possible differences in ill-being may be established over a much longer period of time rather than our brief two-week period. The accumulation of factors such as low vitality, reduced motivation, and poorer socio-emotional flourishing may precipitate the development of psychological ill-being over time [54]. In saying this, Smith and Rogers [24] did find a difference in self–reported anxiety using the same measure (HADS) over a 10-day period with snacking on one piece of fruit each day compared to snacking on chocolate. However, their participants had higher average baseline anxiety (approximately +1.5 points). Intervention studies with positive outcomes tend to have participants with higher baselines of illness [55], which may explain the discrepancy in our findings.

The lack of psychological improvement among the EMI participants is challenging to interpret. Both the EMI and FVI groups reported higher and equivalent consumption of FV compared to control, but the EMI group did not show any corresponding psychological benefit. Both intervention conditions were given roughly the same amount of monetary goods at the start of the study–a $10 voucher for the EMI condition versus a little more than $10 worth of fruit and vegetables for the FVI condition. One possibility is that this difference might be due to lower control over the type, quality, and preparation of fruit and vegetables eaten by the EMI group. The EMI group were free to choose whatever fruit and vegetables they liked, and when surveyed, we found that they were more likely to eat cooked vegetables in casseroles or mixed into their main meals [26]. By contrast, for the FVI group, we chose high quality produce, which was mostly eaten raw (including the carrots, eaten as snacks). Some researchers have shown that more optimal psychological outcomes are associated with the consumption of fresh fruit and raw vegetables/ salads, but not cooked vegetables [56, 57]. It is also possible that regular text-reminders (twice daily for two weeks) might have annoyed participants in the EMI condition and wiped out any gains in well-being. However, our experience is that participant burden is relatively low with receiving only two text messages per day. Furthermore, informal feedback from the EMI participants was mostly positive; participants reported that the messages were not particularly intrusive and were effective at making them more aware about what they ate [26]. Alternatively, it could be that giving FV to participants triggered more intrinsic rather than extrinsic regulation of behavior. A previous study found that households given boxes of free FV were reluctant to waste the food and therefore had to overcome internal conflicts in their personal value systems and find solutions they could live with–contributing to a largely internal source of motivation to consume the FV they had been given [58]. Given that intrinsic goals tend to be more rewarding and lead to greater well-being [59], this could potentially explain the psychological improvement in the FVI condition, but not in the EMI condition.

The significant changes in well-being occurred despite relatively small changes in FV consumption (+ 1 serving per day more than control, and +1.2 servings from baseline for the FVI condition). In fact, participants in the FVI condition still fell short of the “5+ a day” FV servings target [27], let alone the optimal 7–9 daily servings that has been associated with long-term health [1]. Psychological improvements could have been larger had participants in the experimental conditions eaten even more FV (or higher quality FV) or had they achieved saturation levels of vitamin C and carotenoids. As we reported in Brookie et al. [26], the micronutrient increases in vitamin C and carotenoids were modest for our intervention conditions and well-below saturation levels, suggesting that there was still room for improvement. In future research, it would be important to increase consumption more than we did here.

Neither vitamin C and carotenoids nor psychological expectancies about FV mediated the psychological benefits of FV consumption in this study. This was despite us choosing the two most responsive micronutrient biomarkers of FV consumption [32] and measuring a range of psychological expectancies. The literature on micronutrient status and mental health suggests that single nutrients are unlikely to play a large role in the protection against mental illness [60]. Instead, better mental health may arise from the cumulative effects of a broad spectrum of vitamins, minerals, and antioxidants [61]. Future research should consider how FV consumption affects well-being through multiple pathways, including through improved blood flow to the brain [62] or changes in gut microbiota [63, 64].

Strengths of the study included the three-arm randomized design that compared two experimental conditions to a diet-as-usual control condition; use of a smartphone survey to track mood and well-being in near-to-real time which provided a more accurate assessment than retrospective self-report; high compliance with the smartphone survey; and, inclusion of blood samples as an objective marker of FV consumption. Limitations included issues with measurement reliability for some of the diary measures, which was a consequence of efforts to keep the survey short. This could explain why we did not see intervention effects on mood. Future research should include a more extensive measure of mood, such as the 9-item measure of positive mood previously found associated with FV [11, 15]. Another limitation is the short timeframe of our intervention (two weeks) and lack of longer-term follow-up. In this regard, our approach was more of a ‘light touch’ intervention to evoke near-term behavioral change. For this reason, we consider these results preliminary, offering proof of concept that small changes to FV consumption over a short time period may confer changes in well-being.

Our findings have implications for campaigns designed to increase FV consumption. FV campaigns reflect an ‘information is power’ ethos and largely consist of educative programmes operating through child health and wellness services, schools, communities, and public service television ads. However, our research suggests that simply educating people about FV and reminding them to eat their recommended daily intake may not be sufficient in ensuring the wider population reaps the psychological benefits of FV consumption. It is already established that successful interventions tend to be more personal and more intensive [55], but perhaps greater emphasis needs to be placed on actually providing people with fresh FV (stocking more FV in dorms, cafeterias, workplaces, substituting fruit for dessert, and offering free fruit for people when they shop). Additionally, conveying the immediate psychological benefits of FV consumption may have more impact on behavior. Behavioral change messages are more effective when they are immediately relevant to the target population [65] suggesting that near-term benefits–such as increases in vitality and motivation–may be more salient than longer-term health benefits–such as the prevention of heart disease. This may be particularly pertinent to young people, who are endowed with feelings of immortality and quite focused on the ‘here and now’.

## Conclusions

Providing young adults with high-quality FV, not texting them reminders to eat more FV and giving them a voucher, resulted in improvements to their psychological well-being over a two-week period. This is the first study to show that providing high-quality FV to young adults can result in short-term improvements in vitality, flourishing, and motivation. Findings provide initial validation of a causal relationship between FV and well-being, suggesting that large-scale intervention studies are warranted.

## Supporting information

S1 File Pre- and Post-intervention Variables. (SAV)

S2 File Daily diary Variables. (SAV)

S1 Table CONSORT Checklist for Reporting a Randomized Trial. (PDF)

S2 Table Separate Analyses of Mood Items and Flourishing Behaviours. (PDF)

S1 Text Research Protocol. (PDF)
