# Supplementary material for: Let them eat fruit! The effect of fruit and vegetable consumption on psychological well-being in young adults: A randomized controlled trial
Source: PLoS One. 2017 Feb 3;12(2):e0171206. doi: 10.1371/journal.pone.0171206 (PMC5291486; doi:10.1371/journal.pone.0171206)
Supplement: S2 Table — (PDF) [file pone.0171206.s004.pdf]

Supplementary Analyses for Conner, Brookie, Carr, Mainvil, Vissers

Mood

This section presents the results of supplementary analyses testing the three negative mood items (*sad*, *anxious*, *angry*) and three positive mood items (*relaxed*, *happy*, *enthusiastic*) separately. Results are shown in Table S2-a. *Happy* showed significant group differences in change over time (p = .008). Analysis of simple slopes showed that happiness was stable over time for participants in the fruit and vegetable intervention (FVI) condition (B(SE)= -0.001(0.006), p = .845), but decreased for participants in the ecological momentary intervention (EMI) condition (B(SE) = -0.034(0.010), p = 0.001) and control condition (B(SE)= -0.012(0.008), p = 0.018). The slopes were significantly different only between the FVI and EMI conditions (p = 0.008).

Table S2-a. Results from growth curve multilevel modelling testing for changes in the six mood outcomes across the two week intervention period and differences between the control, ecological momentary intervention (EMI), and fruit and vegetable intervention (FVI) conditions. Significant intervention effects are bolded.

Mood Outcome								
Sad	G	Coef	*SE*	*p*		Coef	*SE*	*p*
Control Day 1	G00	0.576	0.079	< 0.001				
EMI Day 1 diff	G01	0.022	0.114	0.849				
FVI Day 1 diff	G02	0.137	0.108	0.204				
Ethnicity	G03	0.027	0.090	0.767				
Control Change	G10	0.013	0.008	0.089				
EMI Change diff	G11	-0.004	0.012	0.760	EMI Δ	0.010	0.009	0.287
FVI Change diff	G12	-0.016	0.011	0.144	FVI Δ diff	-0.012	0.012	0.303
Anxious	G	Coef	*SE*	*p*				
Control Day 1	G00	1.103	0.113	< 0.001				
EMI Day 1 diff	G01	-0.120	0.140	0.391				
FVI Day 1 diff	G02	0.008	0.143	0.955				
Ethnicity	G03	-0.120	0.108	0.269				
Control Change	G10	0.013	0.010	0.175				
EMI Change diff	G11	-0.006	0.015	0.657	EMI Δ	0.007	0.011	0.568
FVI Change diff	G12	-0.015	0.012	0.243	FVI Δ diff	-0.008	0.014	0.563
Angry	G	Coef	*SE*	*p*				
Control Day 1	G00	0.548	0.074	< 0.001				
EMI Day 1 diff	G01	-0.108	0.104	0.303				
FVI Day 1 diff	G02	0.005	0.102	0.965				
Ethnicity	G03	0.023	0.076	0.764				
Control Change	G10	0.006	0.007	0.470				
EMI Change diff	G11	0.006	0.011	0.575	EMI Δ	0.012	0.008	0.135
FVI Change diff	G12	-0.007	0.010	0.466	FVI Δ diff	-0.006	0.011	0.575

Relaxed	G	Coef	*SE*	*p*		Coef	*SE*	*p*
Control Day 1	G00	1.941	0.111	< 0.001				
EMI Day 1 diff	G01	0.323	0.144	0.026				
FVI Day 1 diff	G02	0.108	0.131	0.819				
Ethnicity	G03	-0.067	0.094	0.478				
Control Change (Δ)	G10	-0.018	0.011	0.096				
EMI Change diff	G11	-0.008	0.016	0.606	EMI Δ	-0.026	0.011	0.024
FVI Change diff	G12	-0.009	0.014	0.487	FVI Δ diff	-0.001	0.014	0.929
Happy	G	Coef	*SE*	*p*				
Control Day 1	G00	2.632	0.083	< 0.001				
EMI Day 1 diff	G01	0.072	0.125	0.567				
FVI Day 1 diff	G02	-0.114	0.101	0.262				
Ethnicity	G03	-0.195	0.087	0.026				
Control Change	G10	-0.020	0.008	0.018				
EMI Change diff	G11	-0.014	0.013	0.292	EMI Δ	-0.034	0.010	0.001
FVI Change diff	G12	0.019	0.011	0.082	**FVI Δ diff**	**0.032**	**0.012**	**0.008**
Enthusiastic	G	Coef	*SE*	*p*				
Control Day 1	G00	1.864	0.115	< 0.001				
EMI Day 1 diff	G01	0.206	0.153	0.180				
FVI Day 1 diff	G02	0.035	0.143	0.809				
Ethnicity	G03	-0.145	0.110	0.188				
Control Change	G10	-0.006	0.008	0.453				
EMI Change diff	G11	-0.017	0.014	0.203	EMI Δ	-0.024	0.011	0.025
FVI Change diff	G12	0.008	0.013	0.513	FVI Δ diff	0.026	0.014	0.070

Note. Coef = coefficient from Hierarchical Linear Modeling; SE = Robust standard error; diff = difference in coefficient; EMI = ecological momentary intervention condition; FVI = fruit and vegetable intervention condition; Δ = change. Degrees of freedom were 167 for G00 – G03 and 168 for G10 – G12.

Flourishing Behaviors

This section presents the results of supplementary analyses testing the three flourishing behavior items separately (*curious*, *creative, motivated*). Results are shown below in Table S2-b. Participants in the FVI condition showed significantly different growth patterns in curiosity compared to both the control condition and the EMI condition (G12 coefficients). Analysis of simple slopes showed that curiosity was stable over time for participants in FVI condition (*B*(*SE*)= 0.004(0.008), *p* = .631) but decreased for participants in the control and EMI conditions (control *B*(*SE*) = -0.018(0.008), *p* = 0.018; EMI *B*(*SE*)= -0.026(0.008), *p* = 0.001). The findings for creativity showed a trend difference in the growth trajectories between the FVI and control conditions. Analysis of simple slopes showed that creativity was stable over time for all three groups, but the direction of growth (i.e., the sign of the coefficient) was positive for FVI participants (*B*(*SE*)= 0.013(0.009), *p* = .158), neutral for EMI participants (*B*(*SE*)= 0.001(0.008), *p* = 0.893) and negative for control participants (*B*(*SE*) = -0.006(0.007), *p* = 0.373). Lastly, participants in the FVI group showed significant growth patterns in motivation compared to the control group. Analysis of simple slopes showed that motivation increased over time for participants in the FVI condition (*B*(*SE*)= 0.019(0.010), *p* = .045) but was unchanged for participants in the control and EMI conditions (control *B*(*SE*) = -0.011(0.008), *p* = 0.218; EMI *B*(*SE*)= -0.005(0.011), *p* = 0.652). In terms of effect sizes, being assigned to the FVI condition (vs. control) predicted 10.4% of the variance in the growth in curiosity, 1.8% of the growth in creativity, and 6.0% of the growth in motivation using variance-explained effect size estimates of the time slopes (Raudenbush, & Bryk, 2002).

Table S2-b. Results from growth curve multilevel modelling testing for changes in the three flourishing behavior outcomes across the two week intervention period and differences between the control, ecological momentary intervention (EMI), and fruit and vegetable intervention (FVI) conditions. Significant intervention effects are bolded.

Outcome								
Curious	G	Coef	*SE*	*p*		Coef	*SE*	*p*
Control Day 1	G00	1.490	0.115	< 0.001				
EMI Day 1 diff	G01	-0.014	0.140	0.923				
FVI Day 1 diff	G02	-0.049	0.132	0.706				
Ethnicity	G03	0.040	0.111	0.722				
Control Change (Δ)	G10	-0.018	0.008	0.018				
EMI Change diff	G11	-0.007	0.011	0.490	**EMI Δ**	**-0.026**	**0.008**	**0.002**
FVI Change diff	G12	0.022	0.011	0.043	**FVI Δ diff**	**0.029**	**0.011**	**0.009**
Creative	G	Coef	*SE*	*p*				
Control Day 1	G00	1.205	0.119	< 0.001				
EMI Day 1 diff	G01	-0.247	0.137	0.073				
FVI Day 1 diff	G02	-0.105	0.140	0.457				
Ethnicity	G03	0.067	0.111	0.547				
Control Change	G10	-0.006	0.007	0.373				
EMI Change diff	G11	0.007	0.011	0.499	EMI Δ	0.001	0.008	0.893
FVI Change diff	G12	0.019	0.012	0.097	FVI Δ diff	0.012	0.012	0.344
Motivated	G	Coef	*SE*	*p*				
Control Day 1	G00	1.945	0.116	< 0.001				
EMI Day 1 diff	G01	0.007	0.148	0.964				
FVI Day 1 diff	G02	-0.177	0.141	0.212				
Ethnicity	G03	0.042	0.104	0.683				
Control Change	G10	-0.011	0.008	0.218				
EMI Change diff	G11	0.006	0.014	0.671	EMI Δ	-0.005	0.011	0.652
**FVI Change diff**	**G12**	**0.030**	**0.013**	**0.022**	FVI Δ diff	0.024	0.015	0.098

Note. Coef = coefficient from Hierarchical Linear Modeling; SE = Robust standard error; diff = difference in coefficient; EMI = ecological momentary intervention condition; FVI = fruit and vegetable intervention condition; Δ = change. Degrees of freedom were 167 for G00 – G03 and 168 for G10 – G12.
