# Supplementary material for: Let them eat fruit! The effect of fruit and vegetable consumption on psychological well-being in young adults: A randomized controlled trial
Source: PLoS One. 2017 Feb 3;12(2):e0171206. doi: 10.1371/journal.pone.0171206 (PMC5291486; doi:10.1371/journal.pone.0171206)
Supplement: S1 Text — (PDF) [file pone.0171206.s005.pdf]

UNIVERSITY of OTAGO
Te Whare Wānanga o Otāgo
NEW ZEALAND

H15/010

Academic Services
Manager, Academic Committees, Mr Gary Witte

29 January 2015

Dr T Conner
Department of Psychology
Division of Sciences
Union Place East/Leith Walk

Dear Dr Conner,

I am again writing to you concerning your proposal entitled "**The effect of fruit and vegetable consumption on psychological well-being: An ecological momentary intervention**", Ethics Committee reference number **H15/010**.

Thank you for your letter of 27th January 2025 addressing the issues raised by the Committee.

The Committee notes the provision of a generic Information Sheet outlining the general nature of the study for all participants, as requested.

On the basis of this response, I am pleased to confirm that the proposal now has full ethical approval to proceed.

The standard conditions of approval for all human research projects reviewed and approved by the Committee are the following:

Conduct the research project strictly in accordance with the research proposal submitted and granted ethics approval, including any amendments required to be made to the proposal by the Human Research Ethics Committee.

Inform the Human Research Ethics Committee immediately of anything which may warrant review of ethics approval of the research project, including: serious or unexpected adverse effects on participants; unforeseen events that might affect continued ethical acceptability of the project; and a written report about these matters must be submitted to the Academic Committees Office by no later than the next working day after recognition of an adverse occurrence/event. Please note that in cases of adverse events an incident report should also be made to the Health and Safety Office:

http://www.otago.ac.nz/healthandsafety/index.html

Advise the Committee in writing as soon as practicable if the research project is discontinued.

Make no change to the project as approved in its entirety by the Committee, including any wording in any document approved as part of the project, without prior written approval of the Committee for any change. If you are applying for an amendment to your approved research, please email your request to the Academic Committees Office:

gary.witte@otago.ac.nz

jo.farrondediaz@otago.ac.nz

Approval is for up to three years from the date of this letter. If this project has not been completed within three years from the date of this letter, re-approval or an extension of approval must be requested. If the nature, consent, location, procedures or personnel of your approved application change, please advise me in writing.

Yours sincerely,

Mr Gary Witte
Manager, Academic Committees
Tel: 479 8256
Email: gary.witte@otago.ac.nz

c.c. Professor M W Colombo Head Department of Psychology

Ref:

Human Ethics Committee
Research Ethics (Health) Application Form

Health Research Approval Process

Researchers conducting health research must receive the approval of either:

The University of Otago Human Ethics Committee (Health) or Health and Disability Ethics Committee (HDEC)

Does your study require HDEC review? Please consult the University's research ethics web site or the HDEC web site

Please note if applying to the HDEC, it is a requirement of the Deputy Vice Chancellor (Research and Enterprise) that the approval letter from that ethics committee must be forwarded to the Academic Committees office.

Section 1 - Details of investigators, including student investigators and title of study

1.1 Principal Investigator (University of Otago staff member responsible for project)

Name: Tamlin Conner **Title:** Dr.
Department: Psychology
Email: tconner@psy.otago.ac.nz

Title of Study: The effect of fruit and vegetable consumption on psychological well-being: An ecological momentary intervention

1.2 Co-investigators

Name: Louise Mainvil **Title** Dr.
Department: Human Nutrition
Email: louise.mainvil@otago.ac.nz

Name: **Title**
Department: **Email:**

1.3 Student investigator

Name: Kate Brookie **Level of Study** PhD
Department: Psychology **Email:** broka011@student.otago.ac.nz

Where there are more co-investigators or student investigators, please insert details on a separate sheet

All researchers <u>must</u> complete PART A and PART C.

If your study includes human participants recruited in their capacity as:

- Consumers of health and disability support services or
- Relatives or caregivers of such consumers or
- Volunteers in clinical trials

...you are also required to complete PART B.

PART A

Section 2 – Protocol and summary

2.1 ***A protocol must be attached to this application before submission to the committee. If this protocol has a unique identifier, enter this below.***

Protocol number (if applicable):

Briefly describe and justify the design of your study, including, if appropriate, the power calculation on which the number of participants is based. Provide power calculation if not explicitly stated in the protocol. [<200 words]

The proposed study is a brief two-week intervention (randomized control trial, RCT) to test the influence of fruit and vegetable (FV) consumption on short-term changes in psychological well-being in a healthy, young adult population with low FV consumption. A total of 150 participants ages 18 – 25 will be randomized into three groups: an increased FV group (to be given FV for two weeks); a FV reminder group (to receive mobile text message reminders to eat more FV for two weeks); and a no-intervention control group. This sample size will provide 93% power to detect a one serving difference in FV consumption between the experimental and control groups (at $p < .05$) and 85% power to detect a .3 standard deviation difference in psychological well-being between groups (at $p < .05$). A range of psychological well-being measures (depression, mood) will be measured before, during, and after the intervention, and compared between groups. Additionally, a blood sample will be taken before and after the intervention for analysis of changes in vitamin c and serum carotenoids as biomarkers of FV consumption. The proposed project will involve innovative assessment and intervention delivery methods utilising mobile phone technology to increase FV consumption in naturalistic environments over time.

2.1.1 Briefly and in plain English, what is the principal study question (hypothesis) that the study will test?

You can refer to page numbers of your study's protocol for further detail if you need to. **[<100 words]**

We hypothesize that eating more fruit and vegetables (FV) will improve the psychological well-being of young adults with baseline low FV consumption even in a brief two-week time period. Additionally, the project will investigate the effectiveness of an innovative intervention technique called Ecological Momentary Intervention (EMI) that utilizes mobile phone technology to deliver health interventions in naturalistic settings.

2.1.2 Briefly describe the background for the study (including, where appropriate, brief discussion of previous research), the population from which the sample will be recruited, the inclusion and exclusion criteria and the impact, if any, of the exclusions on the generalisability of results.

You can refer to page numbers of your study's protocol for further detail if you need to.[<200 words]

Research has established an association between greater FV consumption and reduced depression and increased well-being including happiness. This relationship appears to be dose-dependent, with one study showing those who reported seven or eight FV servings also experienced the highest life satisfaction even when controlling for extensive demographic factors and health behaviors. Research from our own laboratory extended these findings, identifying that FV consumption was associated with a broader range of experiences of human flourishing, curiosity, and engagement among young adults. These indicators of well-being increased on days with more FV consumption. However, whether FV *causes* these benefits in psychological well-being remains unknown. We will recruit healthy young adults from the population of students at the University of Otago. Our prior research has indicated that many of them are not meeting a minimum standard of FV consumption. INCLUSION CRITERIA: in the young adult age range (18 – 25), prescreened for being low FV consumers (i.e., no more than an average of two daily servings), and have an Internet enabled mobile phone (expected that a majority of students will be eligible as this technology is becoming nearly ubiquitous in this population, therefore this inclusion criterion should not affect the generalizability of results.) EXCLUSION CRITERIA: taking antidepressant medication, known allergy to any fruits or vegetables. For more details see included Protocol at the end of this application.

2.1.3 Briefly explain how the study will contribute to new knowledge or improve health outcomes.[<100 words]

This project will advance understanding of the role of fruit and vegetables (FV) in psychological functioning, and may provide insight into potential avenues of improving FV consumption of New Zealanders. If the intervention suggests that FV consumption can improve mood and well-being, this may highlight nutrition as a potential route for improving the mood and well-being among a young adult population. Furthermore, this

study will also improve our current knowledge of the effectiveness of technology-based health intervention strategies.

2.1.4 Briefly summarise the Principal Investigator's qualifications and experience relating to conducting studies of this nature. [<200 words]

The principal investigator, Dr, Tamlin Conner, is a leading researcher and expert in mood and well-being, with a specialized interest in mobile technology and health. She has co-edited the *Handbook of Research Methods for Studying Daily Life* (2012), which makes her an expert in the types of assessment proposed in this project. Her ongoing collaboration with the nutrition department provides excellent support for the proposed project, given its large nutritional component. Dr. Conner is currently the PI of the Daily Life Study, a large (n = 1500) interdisciplinary study, which over the last four years has investigated the nutritional underpinnings of well-being. The running of the Daily Life Study means that the operation of mobile technology, nutrition liaison, mood assessment, and blood collection and analysis, is established and expected to operate smoothly. This provides excellent knowledge and support of the current project which will utilize many of the same methods.

2.2 Provide a brief summary of the main ethical issues that you believe your study may raise as well as detailing your approach or strategy for dealing with them. (This information would also normally be reflected in the participant information sheet under the heading *'Is there any risk of discomfort or harm from participation'* **)** [<200 words]

There is a small chance of discomfort during blood draw, and in some cases minor bruising can occur (although generally disappears in about one to two days). To ensure any pain and discomfort is minimized, trained and experienced phlebotomists from the Department of Human Nutrition at the University of Otago will draw the blood.

2.3 Provide the dates on which you plan to commence and conclude your study.

Planned commencement date: 01/02/2015

Planned conclusion date: 31/03/2016

Section 3 - Sponsors

3.1 ***The sponsor is the organisation with overall responsibility for the initiation, management, and financing arrangements of a study.***

Which of the following best describe the sponsor(s) of your study?

☒University of Otago

☐another academic institution

☐collaborative research group

☐district health board (DHB)

☐other government agency

☐pharmaceutical company

☐medical device company

☐other (e.g. non-governmental organisation (NGO), or contract research organisation)

Please specify [ ]

Section 4 - Localities and participants

4.1 At which localities in New Zealand do you intend to conduct your study?

Written support is essential, whether your study is conducted in New Zealand or overseas and should be either attached to this application or forwarded to the Committee once ethical approval has been granted. The locality needs to be aware of the University's protocol, governance and ethical issues.

☒ tertiary education institution

☐ district health board (DHB)

☐ primary health care organisation

☐ private organisation

☐ other – please specify: [ ] Please provide details: [ ]

4.2 Approximately how many participants do you intend to recruit:

In New Zealand?

1-50 [ ] 51-100 101-**150** [X] 151-200 [ ] Over 200 [ ]

Overseas?

1-50 [ ] 51-100 [ ] 101-150 [ ] 151-200 [ ] Over 200 [ ]

Grand total number of participants: 150 participants from New Zealand

Section 5 - Prior review

5.1 Is this application related to one or more previous applications to <u>any</u> ethics committee?

☐yes

☒ no

If yes, explain the relationship, giving the ethics reference number(s) of the previous application(s).

5.2 **Has an application for this study (or a substantially similar study) previously been declined approval by any other ethics committee in New Zealand or overseas?**

☐yes

☒no (go to section 6)

Section 6 – Study Design

6.1 **Is your study:**

☒an intervention study - Go to section 6.1.1

☐an observational quantitative or laboratory study - Go to section 6.1.2

☐a mixed methods study - Use appropriate sections of 6.1

☐a qualitative study - Go to section 6.1.3

6.1.1 **Which of the following best describes your intervention study?**

Blinding:

☒open-label ☐single-blind ☐double-blind

Arms:

☐two-arm ☒ multi-arm

Design:

☒parallel ☐crossover ☐dose-ranging ☐ cluster ☐factorial

Control:

☐placebo-controlled ☐active-controlled ☐ uncontrolled

Randomisation:

☒randomised ☐non-randomised

Aim:

☐superiority ☐equivalence ☐non-inferiority

☒none of the above – explain unsure

6.2 **Indicate whether peer review of the scientific and statistical quality of your study has been obtained from one or more of the following.**

☐the study's funder

☐the study's sponsor

☒senior colleague(s) in the field

☐other - explain

If you have ticked any of the boxes above, briefly describe the peer review process that has been carried out for your study. Evidence of peer review must be attached to this application including responses to any recommended changes. [<200 words]

The PI requested peer review from Dr Anitra Carr (U Otago, Canterbury), who is a leading expert in the role of micronutrients in human health and disease. She has a background in biomedical research and clinical trials of fruit and micronutrients such as vitamin C on mental and physical health. Dr Carr read through the protocol and made several suggestions including (1) the importance of keeping certain fruits refrigerated to preserve the vitamin C and carotenoid levels and (2) requesting that we clarify our statistical controls (such as controlling for average daily exercise and sun exposure, both of which influence mood). Evidence of the peer review is attached.

☐no review

If "no review", explain why your study has not been peer-reviewed.

[<200 words]

6.3 How do you intend to report or disseminate the results of your study?

☒ article(s) in peer-reviewed scientific journals

☒ internal reports

☒ conference presentations

☐ publication on website

☐ other publications

☐ submission to regulatory authorities (e.g. Medsafe, TGA, FDA, EMA)

☒ other – explain Popular media coverage

6.4 Will any restrictions be placed (for example, by your study's sponsor or funder) on the publication of the results of your study?

☐yes

☒no

If yes, briefly describe these restrictions, and explain why they are in place.

[<200 words]

6.5 **Might data generated in your study, but not reported, be made available for use in future research (e.g. for inclusion in an individual data meta-analysis)?**

☐yes

☒no

If so, you should explain this clearly to potential participants.

Which of the following best describes the form in which data generated by your study will **be published, stored, and, if consent for future use has been given, might be made available to other researchers?**

☐identified

☐potentially identifiable

☐partially de-identified

☐de-identified

☒ anonymous

☐other – describe: ☐

Section 7 - Use of human tissue, including blood and other body fluids

7.1 *The use of human tissue in New Zealand is regulated by the Human Tissue Act 2008 and the Code of Health and Disability Services Consumers' Rights 1996.*

Will human tissue be collected and/or used in your study?

☒yes

☐no **If "no" go to Section 8**

7.2 **What types of human tissue will be collected and/or used in your study?** [<100 words]

Two fasting blood samples (5ml each) will be collected using venipuncture by experienced and trained phlebotomists in the Department of Human Nutrition Clinic at the University of Otago.

7.3 **Will your study involve:**

☒human tissue collected from participants during this study? Go to 7.6

☐existing stored human tissue samples?

7.6 **Will any human tissue samples used in your study be imported from outside New Zealand?**

☐yes

☒no

If yes, explain why it is appropriate to use imported human tissue in your study. [<100 words]

7.7 **Briefly explain how human tissue samples will be stored during your study, and how the privacy of donors and participants will be protected.** [<100 words]

Blood samples will be collected, frozen and securely shipped to the Department of Pathology at the University of Otago, Christchurch campus. Secure storage and privacy will be ensured by Margreet Vissers, who will oversee the processing for Vitamin C and serum carotenoids. A unique identifier (ID number) will be assigned to each subject, and blood analyses will use these codes. Information linking codes to identifying information will be stored securely in the psychology department. All data will exist in electronic format and will be retained in a secure password protected server in the Department of Psychology.

7.8 **Will human tissue collected in New Zealand be sent overseas as part of your study?**

☐yes

☒no

If yes, you should explain this clearly to participants.

7.9 **Will the use of all human tissue in your study be in accordance with the informed consent (including consent to future unspecified research) that has been or will be obtained from participants, donors of existing stored human tissue, or other persons entitled to give informed consent under the Human Tissue Act 2008?**

☒ yes go to 7.10

7.10 **Is consent being sought for future unspecified use?**

☐yes – if so specify the general terms of the additional research

☒no

Notes:

i) A new application will need to be submitted to gain approval of any additional research or testing on samples taken for the current study which is outside the scope of the current specified study.

ii) Consent for the particular research project should be obtained separately from the consent for the future unspecified use of the tissue. This could be

achieved by two separate consent forms or a separately detailed statement on the same consent form. Attach consent form(s).

7.11 What types of tests or analyses will be carried out on human tissue as part of your study?

[<100 words]

Participants will be asked to provide a 5ml blood sample collected by venepuncture pre-and post- intervention. These two blood samples per participant will be processed for Vitamin C and serum carotenoids, and possibly other nutritional biomarkers, using standard best-practice principles by Margreet Vissers at the Department of Pathology at the University of Otago (Christchurch Campus). Dr Vissers and her team have extensive experience in biochemical analysis of these biomarkers of fruit and vegetable consumption.

7.12 What will happen to human tissue at the end of your study, or if participants withdraw consent for its use in this study?

☒ disposal

☒ return to donor, whānau, or family member

☐ return to current holder of existing stored human tissue (e.g. a tissue bank)

☐ transfer to another tissue bank

☐ storage by the research team for use in another study

☐storage by the research team as part of a new tissue bank

☐ other

7.13 Briefly explain your answer above.[<100 words]

Participants will have the option of standard disposal of bloods, or to have them returned in order to carry out disposal of their own. Participants can also indicate if they would like their bloods disposed of using a Karakia, in order to respect Māori traditions regarding human tissue.

7.14 Will any human tissue collected or otherwise obtained from participants in this study but not used in the current study be stored and potentially used in unspecified future research? *You should explain this clearly to potential participants.*

☐yes

☒no

Section 8 - Risk of physical harm to participants

8.1 Briefly and in plain English, describe the risks inherent in the procedures to be undertaken by participants in your study and how these risks will be minimised. Including:

- **risk minimisation by use of health questionnaires**
- **participant exclusion criteria**
- **monitoring during procedures**
- **training of research staff and availability of resuscitation equipment if appropriate**
- **use of EEG, ECG, MRI, TMS, FMRI, EMG, radiation, invasive or surface recordings.** *[<200 words]*

With blood sampling using venipuncture, there is risk of discomfort and minor bruising. In order to reduce these risks, bloods will be collected by trained and experienced phlebotomists. There is also a small risk that an individual has an adverse food reaction to the fruit and vegetables provided to them. To minimize this risk, participants will be pre-screened for any known-food allergies and excluded from participating if an allergy were to present a risk to their health.

8.2 Will your study involve the administration of ionising radiation that is not needed for participants' normal clinical management?

☐yes

☒no – go to 8.5

8.5 If this is an intervention study, briefly outline the criteria for its termination, including reference to your study's protocol where appropriate. [<100 words]

In the unlikely event that giving people FV results in psychological harm, we will terminate the intervention. The PI will monitor psychological harm by checking whether pre- and post-test intervention depression scores increase among experimental group participants in the first waves of data collection run in March and April 2015. This is unlikely because prior research shows that healthy eating benefits – not harms – mental health (*Carr et al., 2013, Mood improvement in young adult males following supplementation with gold kiwifruit, a high-vitamin C food, J Nutr Sci; Smith & Rogers, 2014, Positive effects of a healthy snack (fruit) versus an unhealthy snack (chocolate/crisps) on subjective reports of mental and physical health: a preliminary intervention study, Front Nutr*)

Section 9 - Risks to participants other than physical risks of an intervention

9.1 Could participation in the study, or reporting of the findings, risk psychological harm to participants?

☐yes

☒no

If yes, how this risk will be minimised and managed. [<100 words]

9.2 Could participation in the study, or reporting of the findings, risk stigmatising individuals or population groups, or punishment/ harassment for participation?

☐yes

☒no

If yes, how this risk will be minimised and managed. [<100 words]

Section 10 - Risk of potential conflict of interest

10.1 Funding and remuneration

Briefly describe the main source(s) of funding for your study.[<100 words]

University of Otago Research Grant was funded for $14,791 in October 2014. This will allow us to purchase the fruit and vegetables, reimburse participants for their travel costs, and pay for the blood collection and analyses.

10.2 Does the Principal Investigator, any co-investigator, or any direct member of their families have any commercial interest in the intervention(s) to be studied, or any financial relationship to the study sponsor or funder(s), that may inappropriately influence his or her conduct in the study?

☐yes

☒no

10.3 Will the Principal Investigator or any co-investigator be remunerated for their involvement in the study in a way that may inappropriately influence his or her conduct in the study (for instance, bonuses for favourable results or high recruitment rates)?

☐yes

☒no

10.4 Other potential conflicts of interest

Will any researchers in the study face other conflicts of interest (e.g. academic dependence, personal belief)?

☐yes

☒no

10.5 Briefly describe how the risks of any conflict of interest, described in sections 10.1 to 10.4 above, will be minimised and managed.[<100 words]

NA

Section 11 - Risk of breach of privacy and confidentiality

11.1 Before the study:

Will your study involve reviewing or screening health information, for example in order to identify potential participants?

☐yes

☒no

If yes, briefly explain how you will ensure the confidentiality of this health information before the study. [<100 words]

11.2 Will your study involve the use of surveys or questionnaires?

☒yes (copies of the surveys are included at the end of this application)

☐no

Section 12 - Risks to researchers and third parties

12.1 Briefly indicate whether your study may pose any significant risks to researchers and/or third parties, and briefly explain how such risks will be minimised and managed. [<100 words]

N/A

Section 13 - Informed Consent

13.1 Will all participants in your study be competent to, and asked to, provide their informed consent to participate?

☒yes, all participants will be competent to, and asked to give informed consent - If yes go to 13.2

☐no, one or more participants may not be competent to, or will not be asked to give informed consent

13.2 Does the research involve participants giving oral consent rather than written consent?

☐yes

☒no

If yes, explain and justify and incorporate the justification in the Information Sheet.

13.3 **Briefly explain the process by which potential participants in your study will be identified, approached, provided with an Information Sheet written in language appropriate to the intended participants, have the opportunity to ask questions, and be asked to give their informed consent free from undue influence. Identify the person or persons who will conduct the process.**

The student investigator will be in charge of recruiting, identifying, and engaging with potential participants, as well as providing them with an Information Sheet that explains the intervention procedure. Participants will be made aware that they have the right to discontinue the study at any point, or request extra information. Participants will be recruited via advertisements, flyers and word of mouth, as well as through the Experiment Participation pool which operates through the Psychology Department.

13.4 **Will consent be recorded by signature on an individual consent form?**

☒yes

☐no

If no, explain how participants' informed consent will be recorded, and incorporate this information in the Information Sheet.

13.5 **Does the research involve deception, covert observations, or other ways in which information is deliberately withheld or concealed from participants?**

☐yes

☒no

If yes, explain why it is felt appropriate to withhold or conceal information from participants in your study. [<100 words]

13.6 **How will you ensure that participants receive information that becomes available during the study (for example, an unexpected incidence of adverse events in your study, or information from elsewhere) that may be relevant to their continued participation?**

Participants are provided with contact details of both the student investigator and the principal investigator, and are encouraged to contact them if any issue arises. Furthermore, given the mobile phone aspect of the intervention, we are in constant communication with participants should adverse events occur.

13.7 **Will you inform participants of the results of your study?**

☐yes

☒no

Either explain how you will inform participants or explain why you do not intend to do so.

Participants will be given the chance to ask about the research, and told that extra information, including the outcome of the study, will be available upon request. However, the data will not be distributed to all participants due to cost and time constraints.

13.8 Will participants receive any payments, reimbursement of expenses or any other benefits or incentives for taking part in your study?

☒yes

☐no

If yes, describe these, and explain why they are appropriate.

A focus group completed in May 2014 identified cost as a major barrier in preventing students from reaching daily FV requirements. To counteract this barrier, we will supply participants with approximately $10 worth of fruit and vegetables to consume, or a $10 supermarket voucher to offset the costs of consuming FV depending on the experimental condition. For participants recruited by flyers or word of mouth, we will additionally reimburse their travel expenses for coming to our lab twice and completing the daily surveys (up to $40).

13.9 Will you seek consent from participants to inform health practitioners with responsibility for their health care that they are taking part in your study?

☐yes

☒no

Section 14 - Consultation with population groups

Population groups, particularly Māori, should be consulted in the design and conduct of research that is of relevance to them.

14.1 Describe whether and how your study may benefit Māori, and identify the main cultural issues that may arise for Māori who may participate in your study, and explain how these issues will be managed.[<200 words]

Yes, as a matter of collecting full and detailed demographics of our participants, ethnic origin using the 2006 NZ Census categories will be asked in the study along with age, gender, and year at University. Ethnicity will not be used to draw comparisons between ethnic groups – this is not the purpose of the current study. However, the findings of this intervention will benefit the Māori population as the inadequate levels of FV consumption among this age group is present among all ethnicities, including Māori.

14.2 According to the Health Research Council's Guidelines for Researchers on Health Research Involving Māori, is formal consultation with Māori required for your study?

☐yes

☒no

14.3 The University of Otago has a Policy for Research Consultation with Māori. Have you already completed, or do you propose to undertake Māori consultation?

(Please see http://www.otago.ac.nz/research/maoriconsultation/index.html).

☐yes, we have ALREADY undertaken consultation

(attach a copy of your completed Research Consultation with Māori Form)

☒ no - If no, provide a brief outline of reasons why not

An application was submitted on 8 January, 2015. A copy of this submission form is included at the end of this application.

PART C – Signatures

Applicant's signature:
(Principal Investigator)

Name: (please print) TAMLIN S CONNER

Date: 8 JANUARY, 2015

Departmental Approval

Departmental approval: I have read this application and believe it to be scientifically and ethically sound. I approve the research design. The research proposed in this application is compatible with the University of Otago policies and I give my consent for the application to be forwarded to the University of Otago Human Ethics Committee (Health) with my recommendation that it be approved.

Signature of *Head of Department:

Name: (please print) MICHAEL COLOMBO

Date: 12/1/15

***In cases where the Head of Department is also the principal researcher then the appropriate Dean or Pro-Vice-Chancellor must sign.**

UNIVERSITY of OTAGO
Te Whare Wānanga o Otāgo
NEW ZEALAND

<table>
<tr><td>Study title:</td><td colspan="2">Role of Nutrition in Daily Life
(Note: this is the information sheet for the EMI condition)</td></tr>
<tr><td>Principal investigator:</td><td>Name Tamlin Conner
Department of Psychology
Senior Lecturer</td><td>Contact phone number:
479-7624</td></tr>
</table>

Introduction

Thank you for showing an interest in this project. Please read this information sheet carefully. Take time to consider and, if you wish, talk with relatives or friends, before deciding whether or not to participate.

If you decide to participate we thank you. If you decide not to take part there will be no disadvantage to you and we thank you for considering our request.

What is the aim of this research project?

We are attempting to gain an understanding of factors that contribute to variation in young adults' health and well-being. Specifically, we are interested in how various lifestyle factors may contribute to daily experiences of health and vitality. Studies have linked various patterns of eating to aspects of positive and negative psychological well-being. Young adults, notably students, are especially at risk of consuming insufficient vitamins and nutrients associated with foods such as complex carbohydrates, and fruits and vegetables. Results of this study will help inform the relationship between nutrition, health, and psychological factors.

Who is funding this project?

This project is funded by a University of Otago Research Grant.

Who are we seeking to participate in the project?

We are seeking men and women between the ages of 18 and 25 years. In order to be eligible to participate you will need to be willing to provide two blood samples, and have access to a smart phone (with text messaging and internet capabilities). You will have been pre-screened for particular dietary requirements and the use of medications that may interfere with the results of the study. If you are currently using any anti-depressant medication or have any known food allergies please notify your research assistant now.

If you participate, what will you be asked to do?

Should you agree to take part in this project, you will be asked to attend an initial session during which you will complete a computer questionnaire collecting information on your age, gender, ethnicity, and general mood. This first visit will take approximately 20 minutes. During the initial laboratory session you will also receive a $10 grocery voucher to cover any potential costs encountered during the study.

You will also attend two (2) clinic visits at the university over a two week period. A small blood sample (5ml, approximately one teaspoon), will be taken each time by a trained nurse.

The 14 day study will begin the same day as this initial laboratory session. Participants will undergo 'tracking', whereby daily surveys will be sent directly to your mobile phones at 7:00 pm (and must be completed before 1:00 am). The surveys will be sent via hyperlink straight to your mobile phone, only take a few minutes to complete, and include questions about your mood, what you did that day, what you ate, etc.

Participants are expected to follow their normal daily routine, but some participants will be given an extra challenge to think about, and change, what they eat, which will be aided by daily text reminders. If you receive these texts, you are <u>not</u> required to respond.

After the 14 days of filling in these daily-diaries, you will be asked to attend a second lab session to complete another brief 15 minute questionnaire, followed by debriefing. You will receive up to $40 as reimbursement for costs encountered over the course of the study OR if you are a psychology participant, you will receive up to 3 course credits. Completion of at least 10 daily surveys is required to receive this reimbursement in full, in addition to attending all clinic and laboratory sessions.

Is there any risk of discomfort or harm from participation?

You may experience slight pain during the drawing of blood samples. In some cases minor bruising can occur, although this generally disappears in about one to two days. A trained and experienced phlebotomist will draw your blood.

Please know that you can choose to answer only those questions which you feel comfortable answering. Also, tracking your emotions may make you more aware of your emotions, if you are concerned with how you're feeling, there are on-campus counselling services available at Student Health Services.

What specimens, data or information will be collected, and how will they be used?

In laboratory sessions, we will collect demographic information (including age, gender, and ethnicity) and, information about your general well-being. In text messaging and brief surveys delivered to your mobile phones you will be asked questions about various food and drink consumption as well as general mood and well-being. This may include, what you at during a particular day and how your mood was over the past week.

We are collecting blood samples to provide measures of micro-nutrients and overall health. All blood samples will be coded by a study ID number that is not your name. Sample will be stored in a securely locked area within the University of Otago and its affiliated clinic facilities. At all times, the sample will be stored under a code that only the Principle Investigator can link your name, in an anonymous manner. Upon completion your sample will be kept in storage until fully utilized. If any blood should be disposed of during the course of the study, you will have the option to have it disposed of with an appropriate karakia or have it returned to you.

You will be identified by a study ID number, thus your identity will not be known to those conducting the study. Researchers, Tamlin Conner and Kate Brookie will have access to data stored with an assigned ID number only. The data will not be able to be linked back to you. At the end of the project any personal information will be destroyed immediately, except that, as required by the University of Otago's research policy, any raw data on which the results of the research project depend will be retained in secure storage for at least five years.

The results of the project may be published and will be available in the University of Otago Library (Dunedin, New Zealand) with every attempt will be made to preserve your anonymity.

You are most welcome to request a copy of the results following the end of your participation in the study and/or a copy of the results of the project should you wish.

In the event that the surveys ask questions that make your feel hesitant or uncomfortable in any way, you are reminded of your right to decline to answer any particular question(s).

What about anonymity and confidentiality?

Your data will be identified only by a unique study ID number, not your name or mobile phone number. These data will be used for analyses and stored indefinitely – without any personally identifying information – in a password protected location within the servers of the psychology department.

Extra security precautions will be taken to protect the data sent to and from mobile phones and the internet. When you text your reply, the information will be sent to a secure commercial SMS server, where it will be stored and only accessed by the Principal Investigator or nominated associate. The survey will require you to enter a chosen email address and a password to access the survey site. Once you have completed the survey, the information will be automatically encrypted and sent to a secure server housed within the Psychology Department. The data collected will be securely stored on the server until accessed by the Principal Investigator, with assistant from the Psychology Department technician responsible for the server function.

At the end of the project any personal information (contact information, phone numbers etc.) kept on file in the lab will be destroyed. All data, which will exist in electronic format only will be retained in secure electronic storage and archived indefinitely. Again, you name will not be affiliated with these data.

If you agree to participate, can you withdraw later?

You may withdraw from participation in the project at any time and without any disadvantage to yourself.

Any questions?

If you have any questions now or in the future, please feel free to contact either:

**Tamlin Conner**<br>**Principal Investigator**<br>**Department of Psychology**	**Contact details:**<br>**University telephone number: 479-7624**<br>**Email: tconner@psy.otago.ac.nz**
**Kate Brookie**<br>**Research Assistant**<br>**Department of Psychology**	**Contact details:**<br>**University telephone number: 479-9472**<br>**Email: broka011@student.otago.ac.nz**

This study has been approved by the University of Otago Human Ethics Committee (Health). If you have any concerns about the ethical conduct of the research you may contact the Committee through the Human Ethics Committee Administrator (phone +64 3 479 8256 or email gary.witte@otago.ac.nz). Any issues you raise will be treated in confidence and investigated and you will be informed of the outcome.

Study title:	Role of Nutrition in Daily Life<br>(Note: this is the information sheet for the FV and control condition)	
Principal investigator:	Name Tamlin Conner<br>Department of Psychology<br>Senior Lecturer	Contact phone number:<br>479-7624

Introduction

Thank you for showing an interest in this project. Please read this information sheet carefully. Take time to consider and, if you wish, talk with relatives or friends, before deciding whether or not to participate.

If you decide to participate we thank you. If you decide not to take part there will be no disadvantage to you and we thank you for considering our request.

What is the aim of this research project?

We are attempting to gain an understanding of factors that contribute to variation in young adults' health and well-being. Specifically, we are interested in how various lifestyle factors may contribute to daily experiences of health and vitality. Studies have linked various patterns of eating to aspects of positive and negative psychological well-being. Young adults, notably students, are especially at risk of consuming insufficient vitamins and nutrients associated with foods such as complex carbohydrates, and fruits and vegetables. Results of this study will help inform the relationship between nutrition, health, and psychological factors.

Who is funding this project?

This project is funded by a University of Otago Research Grant.

Who are we seeking to participate in the project?

We are seeking men and women between the ages of 18 and 25 years. In order to be eligible to participate you will need to be willing to provide two blood samples, and have access to a smart phone (with text messaging and internet capabilities). You will have been pre-screened for particular dietary requirements and the use of medications that may interfere with the results of the study. If you are currently using any anti-depressant medication or have any known food allergies please notify your research assistant now.

If you participate, what will you be asked to do?

Should you agree to take part in this project, you will be asked to attend an initial session in which you will complete a computer questionnaire collecting information on your age, gender, ethnicity, and general mood. This first visit will take approximately 20 minutes.

You will also attend two (2) clinic visits at the university over a two week period. A small blood sample (5ml, approximately one teaspoon), will be taken each time by a trained nurse. During this initial laboratory session you will be randomly assigned to one of three experimental conditions.

Depending on which group participants are assigned to, you may be given a number of food items (fruit and vegetables, or sugar-free gum) which you are to consume each day over the course of the study. These items will be of no cost to you, but it is important that you try and consume them as instructed. You will be given more specific information about what you are required to consume by your research assistant.

The 14 day study will begin the same day as this initial laboratory session. Participants will undergo 'tracking', whereby daily surveys will be sent directly to your mobile phones at 7:00 pm (and must be completed before 1:00 am). The surveys will be sent via hyperlink straight to your mobile phone, only take a few minutes to complete, and include questions about your mood, what you did that day, what you ate, etc.

After the 14 days of filling in these daily-diaries, you will be asked to attend a second lab session to complete another brief 15 minute questionnaire, followed by debriefing. You will receive up to $40 as reimbursement for costs encountered over the course of the study OR if you are a psychology participant, you will receive up to 3 course credits. Completion of at least 10 daily surveys is required to receive this reimbursement in full, in addition to attending all clinic and laboratory sessions.

Is there any risk of discomfort or harm from participation?

You may experience slight pain during the drawing of blood samples. In some cases minor bruising can occur, although this generally disappears in about one to two days. A trained and experienced phlebotomist will draw your blood.

Please know that you can choose to answer only those questions which you feel comfortable answering. Also, tracking your emotions may make you more aware of your emotions, if you are concerned with how you're feeling, there are on-campus counselling services available at Student Health Services.

What specimens, data or information will be collected, and how will they be used?

In laboratory sessions, we will collect demographic information (including age, gender, and ethnicity) and, information about your general well-being. In text messaging and brief surveys delivered to your mobile phones you will be asked questions about various food and drink consumption as well as general mood and well-being. This may include, what you at during a particular day and how your mood was over the past week.

We are collecting blood samples to provide measures of micronutrients and antioxidants (e.g., vitamin C and serum carotenoids). All blood samples will be coded by a study ID number that is not your name. Sample will be stored in a securely locked area within the University of Otago and its affiliated clinic facilities. At all times, the sample will be stored under a code that only the Principle Investigator can link your name, in an anonymous manner. Upon completion your sample will be kept in storage until fully utilized. If any blood should be disposed of during the course of the study, you will have the option to have it disposed of with an appropriate karakia or have it returned to you.

You will be identified by a study ID number, thus your identity will not be known to those conducting the study. Researchers, Tamlin Conner and Kate Brookie will have access to data stored with an assigned ID number only. The data will not be able to be linked back to you. At the end of the project any personal information will be destroyed immediately, except that, as required by the University of Otago's research policy, any raw data on which the results of the research project depend will be retained in secure storage for at least five years.

The results of the project may be published and will be available in the University of Otago Library (Dunedin, New Zealand) with every attempt will be made to preserve your anonymity.

You are most welcome to request a copy of the results following the end of your participation in the study and/or a copy of the results of the project should you wish.

In the event that the surveys ask questions that make your feel hesitant or uncomfortable in any way, you are reminded of your right to decline to answer any particular question(s).

What about anonymity and confidentiality?

Your data will be identified only by a unique study ID number, not your name or mobile phone number. These data will be used for analyses and stored indefinitely – without any personally identifying information – in a password protected location within the servers of the psychology department.

Extra security precautions will be taken to protect the data sent to and from mobile phones and the internet. When you text your reply, the information will be sent to a secure commercial SMS server, where it will be stored and only accessed by the Principal Investigator or nominated associate. The survey will require you to enter a chosen email address and a password to access the survey site. Once you have completed the survey, the information will be automatically encrypted and sent to a secure server housed within the Psychology Department. The data collected will be securely stored on the server until accessed by the Principal Investigator, with assistant from the Psychology Department technician responsible for the server function.

At the end of the project any personal information (contact information, phone numbers etc.) kept on file in the lab will be destroyed. All data, which will exist in electronic format only will be retained in secure electronic storage and archived indefinitely. Again, you name will not be affiliated with these data.

If you agree to participate, can you withdraw later?

You may withdraw from participation in the project at any time and without any disadvantage to yourself.

Any questions?

If you have any questions now or in the future, please feel free to contact either:

**Tamlin Conner**<br>**Principal Investigator**<br>**Department of Psychology**	**Contact details:**<br>**University telephone number: 479-7624**<br>**Email: tconner@psy.otago.ac.nz**
**Kate Brookie**<br>**Research Assistant**<br>**Department of Psychology**	**Contact details:**<br>**University telephone number: 479-9472**<br>**Email: broka011@student.otago.ac.nz**

This study has been approved by the University of Otago Human Ethics Committee (Health). If you have any concerns about the ethical conduct of the research you may contact the Committee through the Human Ethics Committee Administrator (phone +64 3 479 8256 or email gary.witte@otago.ac.nz). Any issues you raise will be treated in confidence and investigated and you will be informed of the outcome.

UNIVERSITY of OTAGO
Te Whare Wānanga o Otāgo
NEW ZEALAND

The Role of Nutrition in Daily Life

Principal Investigator: Dr. Tamlin Conner (tconner@psy.otago.ac.nz; (3) 479-7624)

CONSENT FORM FOR PARTICIPANTS

I have read the Information Sheet concerning this project and understand what it is about. All my questions have been answered to my satisfaction. I understand that I am free to request further information at any stage.

I know that:

1. My participation in the project is entirely voluntary.

2. I am free to withdraw from the project at any time without any disadvantage.

3. The questionnaire data will exist in electronic format and will be retained in a secure password-protected server in the psychology department. My name will not be identified with any of the data. I understand that security precautions have been taken to protect data transmitted by texting and the internet.

4. My blood samples will be processed at the University of Otago Christchurch campus for various nutrition-related micronutrients including but not limited to vitamin C and serum carotenoids. The blood results will be used by the Principal Investigator (T. Conner) and her affiliated collaborators to examine hypotheses about the relation between micronutrients and psychological well-being.

   If any blood should be disposed of during the course of the study, please indicate the following:

   CHOOSE ONE:
   - □ I consent to any remaining samples being disposed of using standard disposal methods.
   - □ I wish to have any remaining samples disposed of with appropriate karakia (blessing).
   - □ I wish to have any remaining samples returned to me.

5. In terms of risks, I am aware that I may experience slight pain during the drawing of blood, and in some cases minor bruising may occur but this will generally disappear in about one to two days.

6. My opportunity for compensation may be fewer than 3 credits (or $40) if I miss more than four days of surveys, do not attend the laboratory or clinic sessions, or do not complete the study. Funding is provided by the University of Otago research Grant. The data will be used for scientific purposes only, not for commercial purposes.

7. The results of the project may be published and available in the library with every attempt made to preserve my anonymity.

I agree to take part in this project.

....

(Signature of participant) (Date)

....

(Name of participant)

This study has been approved by the University of Otago Human Ethics Committee (#_____). If you have any concerns about the ethical conduct of the research you may contact the Committee through the Human Ethics Committee Administrator (ph 03 479 8256). Any issues you raise will be treated in confidence and investigated and you will be informed of the outcome.

(Advertisement Flyer) The Role of Nutrition in Daily Life

We are currently recruiting for males and females aged 18-25 to take part in a research study to understand the factors that contribute to the well-being of young adults. All you need to do is complete a questionnaire at an initial laboratory session, answer a daily survey sent directly to your smart phone for 14 days, give two small blood samples, and then come back to complete a final questionnaire. Some participants may be challenged to change the way they eat, and will be provided with certain food items to consume over the course of the study. Some people may not be able to participate if they are currently on anti-depressant medication or have a known fruit and vegetable allergy. If you choose to participate you will be reimbursed up to $40, or up to 3 course credits.

For further information please contact Dr. Tamlin Conner (tconner@psy.otago.ac.nz; 479-7624).

This project has been reviewed and approved by the University of Otago Human Ethics Committee, (Health). Reference: ##/###

The effect of fruit and vegetable consumption on psychological well-being: An ecological momentary intervention

Research Protocol

Introduction

Aim

The primary aim of this project is to conduct a brief 14-day intervention to test whether increased fruit and vegetable (FV) consumption results in improvements in psychological well-being in a young adult population. We have chosen to focus on this age group as they have one of the poorest rates of FV consumption, and very few young adults reach the minimum recommended daily intake (RDI) of 5 servings/day. Overall, the goal is to understand whether our intervention increases FV consumption, whether that increase in FV consumption translates into noticeable short-term improvements in psychological well-being (better mood, lower depression), and whether such changes in well-being are mediated by increased levels of micronutrients derived from FV. If the results suggest FV consumption can promote psychological well-being, this may highlight nutrition as a potential route for improvement in mental states.

Background

The benefits of healthy eating on physical well-being are well established (Bazzano, 2006). Greater fruit and vegetable (FV) intake is associated with a range of positive health outcomes including greater longevity (Bellavia, Larsson, Battai, Wolk, & Orsini, 2013) and reduced risk of potentially fatal disease (Bazzano, 2006; He, Nowson, Lucas, & MacGregor, 2007; He, Nowson, & MacGregor, 2006). There is also growing interest in the psychological benefits of healthy eating, notably the contribution of FV consumption (Rooney, McKinley, & Woodside, 2013). Research has established an association between greater FV consumption and reduced incidence of psychological illnesses including depression (Allgower, Wardle, & Steptoe, 2001; Jacka et al., 2010; McMartin, Jacka, & Colman, 2013), as well as positive indicators of psychological well-being, such as greater happiness (Blanchflower, Oswald, & Stewart-Brown, 2012; Piqueras et al., 2011), higher life satisfaction (Blanchflower et al., 2012; Grant, Wardle, & Steptoe, 2009), dispositional optimism (Giltay, Geleijnsem Zitman, Buijsse, & Kromhout, 2007), and improved self-esteem and self-efficacy (Mainvil, Lawson, Horwath, McKenzie, & Reeder, 2009; Shaikh, Yaroch, Nebeling, Yeh, & Resnicow, 2008). Furthermore, research has found a dose-response correlational relationship between daily servings of FV and life satisfaction, whereby those who consumed 7 or 8 servings of FV per day experienced the highest life satisfaction and happiness (Branchflower et al., 2012). These associations remained significant when controlling for extensive demographic factors such as employment, income, social class, education level, and other health behaviours (e.g. smoking, BMI). Research from our lab extended these findings, identifying that FV consumption was associated with a broader range of experiences of human flourishing including curiosity, meaning, and engagement among young adults (Conner, Brookie, Richardson, & Polak, 2014; White, Horwath, & Conner, 2013).

There is growing evidence to suggest that FV may play a part in helping to prevent ill-being, in addition to promoting positive aspects of well-being. However, whether FV directly *causes* these benefits in psychological well-being is unknown. The majority of research linking FV to well-being is correlational, which cannot establish cause and effect. There are only two intervention studies we have found, but the sample sizes were small. The first study was run at the University of Otago Medical School in Christchurch and found that eating 2 kiwifruit a day for six weeks produced significant improvements in psychological well-being, including liveliness and cheerfulness (Carr, Bozonet, Puller, & Vissers, 2013), compared to those who ate fewer servings. Vegetable consumption was not tested and participants were all male. The second study investigated the effects of healthy and unhealthy snacking on psychological well-being over a brief 10 day intervention period (Smith & Rogers, 2014). The results showed that snacking on one piece of fruit a day significantly reduced anxiety from baseline to follow up compared to snacking on crisps and chocolate. Again, vegetable consumption was not addressed, and well-being was not assessed. However, this latter study suggests that even short-term increases in fruit consumption have noticeable improvements in mental states, at least anxiety.

Rationale

Aside from these two studies, the evidence for a causal effect of FV on well-being is weak. In fact, of the four recent published correlational studies, which included large population studies in both the UK and Australia, each article called for the need for well designed, experimental studies, specifically RCTs. Thus, the primary aim of this project is to conduct an RCT to test whether increased FV consumption results in improvements in psychological well-being over a two-week period. This intervention will target a particularly 'at risk' group – the young adult population aged 18 to 25. This group shows some of the lowest consumption of both fruit and vegetables compared to most age groups (Krebs-Smith, Guenther, Subar, Kirkpatric, & Dodd, 2010; University of Otago and Ministry of Health, 2011).

The proposed project will also pilot an innovative *Ecological Momentary Intervention (EMI)* technique that uses mobile phones to increase FV consumption in naturalistic environments over time. EMIs deliver intervention messages and reminders to individuals as they go about their daily lives. By having instant access into the natural environment of each participant, EMIs are an ecologically valid and effective way to change health behaviour compared to traditional forms of intervention (Heron & Smyth, 2010). Prior EMI research with similar health behaviours (smoking, drinking) have shown EMIs to be effective and are therefore likely to result in increased FV consumption in the proposed study (Heron & Smyth, 2012). With mobile phone use becoming close to ubiquitous, especially among a young population, our intervention will be delivered seamlessly into the daily lives of young adults with the aim of increasing FV consumption. This project will also use mobile phones to measure self-reported FV consumption and psychological well-being throughout the course of the intervention. Real-time survey measures avoid one of the key limitations in the current FV and well-being literature-- an over-reliance on retrospective questionnaires that introduces memory bias to reports (Conner & Barrett, 2012; Schwarz, 2012).

Additionally, this project will investigate the potential biological mechanisms that might account for why FV improves psychological well-being. The literature provides minimal information about the biological mechanisms that may explain this relationship. While some micronutrients and biomarkers, such as C-reactive protein, Vitamin D and Vitamin E have previously been investigated in the literature; there are recent studies suggesting that they *do not* play a significant role in the relationship between FV consumption and psychological well-being (Vitamin D, Polak, 2014; CRP, Thompson & Conner, 2014). Alternatively, Vitamin C and serum carotenoids have been highlighted as potential mechanisms (Boehm et al., 2013; Carr et al., 2013; Rooney et al., 2012), and will be investigated in the proposed study.

Research Questions and Hypotheses

The following research questions and hypotheses have emerged from the findings of our previous studies as well as the gaps identified in the current state of literature.

1. Is FV consumption amongst a 'low consumption' young adult population able to be increased using naturalistic intervention strategies such as EMI?
    a. Hypothesis: Naturalistic intervention strategies such as EMI will successfully increase the daily consumption of FV in this population by at least one daily serving.

2. If these intervention strategies are successful, does the resulting increase in FV improve the psychological well-being (anxiety, depression, creativity, and vitality), of this population?
    a. Hypothesis: Daily increases in servings of FV will result in improved psychological well-being amongst 'low FV consumers' in a young adult population.

3. Is the improvement in psychological well-being (as a result of increased FV consumption) mediated by the micronutrient levels of Vitamin C and Serum Carotenoids in the blood?
    a. Hypothesis: Psychological well-being amongst low FV consuming young adults will improve as a result of increased FV consumption, and this relationship will be mediated by levels of Vitamin C and Serum Carotenoids found in the blood.

Methods

Design

The proposed study is a micro-longitudinal, randomized controlled trial (RCT) investigating the influence of FV consumption on psychological well-being in a young adult population whose FV

consumption is 'low'. This design will allow further investigation of whether the relationship between FV consumption and psychological well-being is causal. Prior to the commencement of the main intervention, a pilot study, of similar design, will be conducted. This will test the effectiveness of the EMI strategy in increasing FV consumption and provide a chance to inform any adjustments that need to be made prior to the main intervention.

Participants

We aim to recruit a representative sample of approximately 150 Dunedin-based students from the University of Otago who are considered low-FV consumers (no more than approximately 2 servings of FV a day). This sample size will provide 93% power to detect a one serving difference in FV consumption between the experimental and control group (at $p < .05$).

Participants will be recruited in three ways. The majority will be recruited from the Department of Psychology experiment participation pool. These participants will be reimbursed with course credit. Additionally, we can use the previous participant pool from the Daily Life Study conducted by Dr Tamlin Conner in 2013 and 2014, and identify those participants who are 'low FV consumers'. These people will be invited to participate in the current study via email. Finally, participants will also be recruited through flyers and advertisements around university. Participants who are not enrolled in a psychology course will receive up to NZ$40 and $10 grocery vouchers to reimburse them for their time and any potential fruit and vegetable purchases.

To be eligible for this study, participants must be in the 'young adult' age range (18-25 years), at least part-time University of Otago students, and own a smartphone (roughly 60% of student body). As this study aims to recruit 'low FV consumers', all participants will be asked about their current state of FV consumption, and only those who register as 'low' (i.e., approximately 2 daily servings) will be able to participate. Those on anti-depressants or with a known FV food allergy will also be excluded from the study.

Procedure

Once the participants are identified, they will be randomly assigned to one of three conditions: Ecological Momentary Intervention (EMI) condition, Fruit and Vegetable (FV) condition, or control. They will then undergo several study components: initial laboratory session, tracking and intervention, follow up laboratory session, and blood sampling. These are described in detail below:

Initial Laboratory Session

Participants will be asked to attend an initial laboratory session during which pre-test measures of psychological health (depression, anxiety, happiness, and vitality) will be completed and a baseline blood sample will be taken by a trained phlebotomist. Some demographic information will also be collected, including age, gender, and ethnicity using NZ census categories. Participants will be randomly assigned to one of three conditions (50 in each group): EMI group, FV group, and control. Once randomly assigned to a condition, each participant will receive an information sheet specific to that condition, as to not confuse them with information about the requirements of the other conditions. However, all conditions will receive basic information about how to accurately report standard FV serving sizes in the daily surveys, embedded amongst other information.

<u>EMI Condition</u>. Those randomly assigned to the EMI intervention group will receive information about the health benefits of FV consumption, including information that they can take home with them. These participants will also be given a 'challenge' to increase their FV consumption, with helpful suggestions for ways to make these changes. For example, those in this condition may receive helpful ideas for FV based snacks, or ways to 'sneak' more FV into evening meals. During this initial session, EMI participants will be given a $10 grocery voucher to control for the FV condition being given approximately $10 worth of fruit and vegetable produce.

<u>FV Condition</u>. During this initial session, those randomly assigned to the FV condition will be given a FV package containing the equivalent to approximately three servings of FV for each day of the 14-day intervention, including both fresh and frozen options. FV choices will be made on what is seasonal, commonly available and what will remain freshest for longest (e.g., carrots, kiwifruits, frozen vegetable medley). All fruit will be kept refrigerated to preserve its nutritional constituents. Participants in the FV condition will be asked to consume these daily servings over the duration of the study.

Control Condition. Those randomly assigned to the control condition will receive no pre-test information, but will receive information on how to accurately report FV serving sizes (embedded among other instructions). This group will receive a 14-pc packet of sugar-free chewing gum, and will be asked to consume one piece each day for the duration of the study. This is to ensure that all conditions are receiving something (FV, text messages, or chewing gum), and are required to engage in the study by remembering to consume these goods each day. During this initial session, control participants will be given a $10 grocery voucher to control for the FV condition being given approximately $10 worth of fruit and vegetable produce.

Tracking and Intervention

Over the following 14 days, all participants will complete a brief daily survey delivered straight to their mobile phones regarding their FV and other food consumption, and measures of vitality and daily mood states. The survey will be delivered as a web hyperlink to participants' mobile phones; clicking on this hyperlink will bring up an online questionnaire asking about their food and drink consumption and their well-being for that day (mood, happiness, engagement, curiosity). The food consumption questions will be based on questions from the New Zealand Adult Nutrition Survey assessing daily servings of fruit (fresh, frozen, canned or stewed; not juice or dried fruit), vegetables (fresh, frozen or canned; not juice or hot chips), and several unhealthy foods for comparison (sweets, crisps, etc; Ministry of Health, 2003). This survey will be completed after the last meal of each day, and will be available to the participants between the hours of 7:00pm and 1:00am. They will receive a text message at approximately 9:00pm to remind them to complete their daily survey. All participants will be briefed on how to access and complete this survey, including a practice run, during the initial laboratory session.

Additionally, during this 14 days of tracking, those in the EMI condition will receive daily text-messages that provide information, prompts, and suggestions for ways to improve their FV consumption in real-time. The texts will be delivered at times relevant to various food choices. For example, a text may be sent on a Tuesday afternoon reminding the participants to "Stop by the Hinton's Stand or Veggie Boys to pick up cheap FV for dinner." Other messages may ask whether they are on track with their goal, such as "How many servings of fruit/veg are you up to today?". They are not required to respond to these texts

Follow-up laboratory session

At the end of the 14 day tracking all participants will complete their second blood sample clinic session and attend a follow-up debriefing session. Participants will be asked to complete a final questionnaire that will include post-test measures of psychological health (depression, anxiety, happiness, creativity and vitality) followed by debriefing. This session should be no longer than 15 minutes long, but research assistants will be available for longer in order to allow participants to ask questions about the study.

Blood Sampling

Participants will provide two morning fasting blood samples - one at baseline prior to the initial laboratory session and one prior to the follow-up laboratory session. During these sessions a small blood sample (5ml) will be taken each time and processed for vitamin c and serum carotenoids. Blood will be drawn by trained phlebotomists in the Department of Nutrition. Samples will be identified by a study number only and disposal with Karakia will be offered to all participants as stated in the consent form. Once the blood is taken it will be frozen and securely stored before being shipped to the Department of Pathology at the University of Otago, Christchurch Campus. Margreet Vissers will oversee the process of blood analyses, and will ensure the storage safety and confidentiality of the samples during this analysis.

Statistical Analysis

Data will be analysed using multilevel modelling to take into account the nested nature of the data. Analyses will test for differences between both the intervention groups and the control group on: pre- and post-test changes in psychological health, average daily differences in vitality and mood; average daily differences in FV consumption. Control variables will include average daily self-reported exercise and average daily time outside (as a marker of sun exposure). The effectiveness of the EMI and FV conditions in improving FV consumption will be tested by comparing changes in these groups

versus control. If either of the intervention groups show higher FV consumption and greater psychological well-being than the control group, we will use mediation analysis to determine whether levels of micronutrients from blood samples explain the differences in psychological well-being between the experimental and control groups. Overall, the goal is to understand whether our intervention increases FV consumption, whether that increase in FV consumption translates into noticeable improvements in psychological well-being (better mood, lower depression), and whether such changes in well-being are mediated by levels of micronutrients derived from FV.

Improving Efficacy and Reducing Demand Characteristics

Published research from our lab suggests that, with training, students of this age group are able to report with reasonable reliability their daily servings of FV (Conner et al., 2014, White et al., 2013). However, as the proposed study is heavily reliant on self-report, it is important to incorporate design features that encourage accurate reporting. In an initial session, participants will get taught how to complete the food consumption questions including portions and serving sizes. We will stress that reporting accurate behaviour is more important than reporting desirable behaviour, and that the study is non-judgmental. Furthermore, the blood analysis for vitamins and carotenoids may provide some information about the current state of healthy eating. Participants will be informed that the inclusion of this blood sample will allow insight into their general health, including healthy vs. unhealthy consumption. This provides a means of promoting accurate reporting based on social psychological principles (which show greater honesty in reporting when their reports can be checked against an objective marker).

Reporting

A report of this study will be written as a part of the student investigator's thesis and as an article for publication in a relevant scientific journal. Additional reporting may include: in department presentations, press releases, and online publications.

Impact on Health Outcomes

The proposed study will likely have a number of positive outcomes in regards to improving the current understanding of nutrition in psychological well-being, as well as potential larger scale influences on health service delivery.

A substantial number of New Zealanders have less than adequate FV consumption, and younger people are especially at risk for unhealthy eating habits (University of Otago, Ministry of Health, 2011). It is not yet known whether the relationship between FV consumption and psychological well-being is causal. This study will be amongst the first in New Zealand to test this relationship at an experimental level, and the first study to investigate both fruit <u>and</u> vegetables. Furthermore, the use of real-time assessment and intervention will draw conclusions on the effects of FV consumption on a daily basis. The results of this intervention will help advance our understanding of the role of FV in daily psychological functioning, and may provide insight in to potential avenues of improving FV consumption in students.

This research will extend our previous understanding of how FV consumption impacts psychological well-being. The results of this study will also provide insight into potential intervention strategies to promote healthy eating, especially among 'high-risk' groups such as young adults. Collectively, this data will help inform organisations such '5+ a Day' which work towards promoting healthy eating. Providing evidence of both physical and psychological benefits of healthy eating may encourage New Zealanders to improve their diet. If the results suggest psychological well-being is being promoted through FV consumption, this may highlight nutrition as a potential route for prevention and treatment of mental illness such as depression.

The data provided by this intervention may provide pilot data to apply similar EMI strategies to other populations who are 'at risk' or who may particularly benefit from increasing their FV consumption. Such populations may include overweight or elderly, and those with depression or high stress loads.

This study will also improve our current knowledge of the effectiveness of technology-based intervention strategies. According to the World Health Organisation (WHO), the near ubiquity of mobile phone use in all areas of the population and increasing mobile functionality (e.g. smart phones, 'apps'), has the potential to "transform the face of health service delivery across the globe". Mobile health services are a vastly growing field, and more research on their efficacy is needed. Thus, it is vital to consider the effectiveness of a mobile phone-based intervention as a cost-effective and

accessible means of promoting FV consumption in all strata of population, and to ultimately strengthen the delivery of health services.

References

Allgöwer, A., Wardle, J., & Steptoe, A. (2001). Depressive symptoms, social support, and personal health behaviors in young men and women. *Health Psychology*, *20*(3), 223-227.

Bazzano, L. A. (2006). The high cost of not consuming fruits and vegetables. *Journal of the American Dietetic Association*, *106*, 1364-1368.

Bellavia, A., Larsson, S. C., Bottai, M., Wolk, A., & Orsini, N. (2013). Fruit and vegetable consumption and all-cause mortality: a dose-response analysis. *The American Journal of Clinical Nutrition*, *98*(2), 454-459.

Blanchflower, D. G., Oswald, A. J., & Stewart-Brown, S. (2012). Is psychological well-being linked to the consumption of fruit and vegetables? *Social Indicators Research*, *114*, 785-801.

Carr, A. C., Bozonet, S. M., Pullar, J. M., & Vissers, M. (2013). Mood improvement in young adult males following supplementation with gold kiwifruit, a high-vitamin C food. *Journal of Nutritional Science*, *2*, 1-8.

Conner, T. S., & Barrett, L. F. (2012). Trends in ambulatory self-report: the role of momentary experience in psychosomatic medicine. *Psychosomatic medicine*, *74*, 327-337.

Conner, T. S., Brookie, K. L., Richardson, A. C., & Polak, M. A. (2014). On carrots and curiosity: eating fruit and vegetables is associated with greater flourishing in daily life. *British Journal of Health Psychology*.

Esmaillzadeh, A., Kimiagar, M., Mehrabi, Y., Azadbakht, L., Hu, F. B., & Willett, W. C. (2006). Fruit and vegetable intakes, C-reactive protein, and the metabolic syndrome. *The American Journal of Clinical Nutrition*, *84*, 1489-1497.

Giltay, E. J., Geleijnse, J. M., Zitman, F. G., Buijsse, B., & Kromhout, D. (2007). Lifestyle and dietary correlates of dispositional optimism in men: The Zutphen Elderly Study. *Journal of Psychosomatic Research*, *63*(5), 483-490.

Grant, N., Wardle, J., & Steptoe, A. (2009). The relationship between life satisfaction and health behavior: a cross-cultural analysis of young adults. *International Journal of Behavioral Medicine*, *16*(3), 259-268.

Hamer, M., and Chida, Y. (2011). Life satisfaction and inflammatory biomarkers: The 2008 Scottish Health Survey. *Japanese Psychological Research 53 (2),* 133–139

He, F. J., Nowson, C. A., & MacGregor, G. A. (2006). Fruit and vegetable consumption and stroke: meta-analysis of cohort studies. *Lancet, 367*, 320-326.

He, F. J., Nowson, C. A., Lucas, M., & MacGregor, G. A. (2007). Increased consumption of fruit and vegetables is related to a reduced risk of coronary heart disease: meta-analysis of cohort studies. *Journal of Human Hypertension, 21*, 717-728.

Heron, K. E., & Smyth, J. M. (2010). Ecological momentary interventions: Incorporating mobile technology into psychosocial and health behavior treatments. *British Journal of Health Psychology, 15,* 1-39.

Hiles, S. A., Baker, A. L., de Malmanche, T., and Attia, J. (2012). Interleukin-6, C-reactive protein and interleukin-10 after antidepressant treatment in people with depression: a meta-analysis. *Psychological Medicine, 42*, 2015–2026

Jacka, F. N., Pasco, J. A., Mykletun, A., Williams, L. J., Hodge, A. M., O'Reilly, S. L., ... & Berk, M. (2010). Association of Western and traditional diets with depression and anxiety in women. *American Journal of Psychiatry*, *167*(3), 305-311.

Krebs-Smith, S. M., Guenther, P. M., Subar, A. F., Kirkpatric, S. I., & Dodd, K.. W. (2010). Americans do not meet federal dietary recommendations. *The Journal of Nutrition, 140*(10), 1832-1838.

Mainvil, L. A., Lawson, R., Horwath, C. C., McKenzie, J. E., & Reeder, A. I. (2009). Validated scales to assess adult self-efficacy to eat fruits and vegetables. *American Journal of Health Promotion, 23*, 210-217

McMartin, S. E., Jacka, F. N., & Colman, I. (2013). The association between fruit and vegetable consumption and mental health disorders: Evidence from five waves of a national survey of Canadians. *Preventive Medicine*, *56*(3), 225-230.

Ministry of Health. (2003). Food and nutrition guidelines for healthy adults: a background paper. Wellington, N.Z.: MOH.

Piqueras, J. A., Kuhne, W., Vera-Villarroel, P., van Straten, A., & Cuijpers, P. (2011). Happiness and health behaviours in Chilean college students: A cross-sectional survey. *BMC Public Health*, *11*(1), 443-453.

Rooney, C., McKinley, M. C., & Woodside, J. V. (2013). The potential role of fruit and vegetables in aspects of psychological well-being: a review of the literature and future directions. *The Proceedings of the Nutrition Society 72 (4),* 420-432.

Schwarz, N. (2012). Why researchers should think "real-time": A cognitive rationale. *Handbook of research methods for studying daily life* (pp. 22-42). M. R. Mehl, & T. S. Conner (Eds.). Guilford Press.

Shaikh, A. R., Yaroch, A. L., Nebeling, L., Yeh, M. C., & Resnicow, K. (2008). Psychosocial predictors of fruit and vegetable consumption in adults: a review of the literature. *American Journal of Preventive Medicine, 34*, 535-543.

Thompson, L. & Conner, T. (2014). *Fruit and vegetable intake and well-being: A relationship mediated by C-Reactive Protein?* Honours Research Thesis, Department of Psychology, University of Otago, Dunedin, New Zealand.

University of Otago and Ministry of Health. (2011). *A Focus on Nutrition: Key findings of the 2008/09 New Zealand Adult Nutrition Survey.* Wellington, NZ: Ministry of Health.

White, B. A., Horwath, C. C., & Conner, T. S. (2013). Many apples a day keep the blues away–Daily experiences of negative and positive affect and food consumption in young adults. *British Journal of Health Psychology, 18*(4), 782-798.

Evidence of Peer Review

From: Anitra Carr [mailto:anitra.carr@otago.ac.nz]
Sent: Monday, 5 January 2015 10:50 a.m.
To: Tamlin Conner
Cc: Margreet Vissers
Subject: Request for "Peer Review"

Dear Dr Conner,

Thank you for the opportunity to review your study design on 'The effect of fruit and vegetable consumption on psychological well-being'. This appears to be a well-designed and timely study – as you point out, very few appropriately design clinical trials have been carried out to address the effect of fruit and vegetable consumption on subjective mood (I have attached a copy of the study protocol with a few minor suggestions).

The use of a mobile phone app is an innovative method for intervening with reminders and also collecting timely and hence more accurate data. The use of two different treatment groups and a control group appears appropriate and the inclusion of vitamin C and carotenoid measurements will help to assess compliance and also begin to address potential mechanisms of action.

The sample size appears to be appropriate, although it is not clear what data was used to carry out the power calculations (presumably this was from a pilot study?). As far as blood sampling is concerned, you may want to state that the samples will be identified by study number only and disposal with Karakia will be offered to Maori participants (this will need to be stated in their consent form). As far as statistical analysis goes, it is not clear what potential confounders you will assess in your study population.

As you have had previous experience carrying out comparable mood food studies and have the necessary resources available through the University, this study appears to be not only suitable, but also feasible.

All the best,

Anitra Carr

Anitra C. Carr, Ph.D.
Senior Research Fellow & Centre Co-ordinator

Department of Pathology, University of Otago, Christchurch
Street: 2 Riccarton Ave, Christchurch 8011, New Zealand

Postal: PO Box 4345, Christchurch 8140, New Zealand

Tel +64 3 364 0649; Mob +64 27 419 1629

Email anitra.carr@otago.ac.nz; http://www.freeradical.org.nz

UNIVERSITY of OTAGO
Te Whare Wānanga o Otāgo
NEW ZEALAND

The effect of fruit and vegetable consumption on psychological well-being

Principal Investigator 1

Name: Dr Tamlin Conner
Department: Department of Psychology
Campus: DUNEDIN
Email: tamlin.conner@otago.ac.nz **Telephone:** Not Supplied

Is this Otago District Health Board research?

No

Does this research involve human participants?

Yes

Description in lay terms of the proposed research

The primary goal of this research is to conduct an intervention to increase fruit and vegetable (FV) consumption among a 'low consuming' young adult population. The proposed project will compare an innovative Ecological Momentary Intervention (EMI) technique that uses mobile phones to increase FV consumption in naturalistic environments over time, versus providing participants with daily servings of FV, versus a no-intervention control group. Participants will report information about their daily mood and food intake over two weeks. Demographic information (including ethnicity based on NZ census categories) will be collected, and blood samples will be taken to measure micronutrient levels both pre- and post-intervention.

Description in lay terms of the potential outcomes of the area of research

Overall, the goal is to understand whether our intervention increases FV consumption, whether that increase in FV consumption translates into noticeable improvements in psychological well-being (better mood, lower depression), and whether such changes in well-being are mediated by levels of micronutrients derived from FV (as measured in the blood). The results of this project will contribute to the understanding of the effects of healthy eating on psychological well-being, and inform strategies to improve FV consumption.

Potential areas that are of interest to or of concern for Māori

There is growing interest in Māori mental and physical health, especially among a young adult population. If we can increase FV consumption among young adults (and show positive effects on mental health), this intervention could potentially be used in future research among Māori young adults with low FV consumption. Demographic information regarding ethnicity

will be collected using New Zealand census categories to ensure that this information is captured. In order to respect Māori traditions regarding human tissue, all participants can indicate if they would like their blood samples returned to them or disposed of using a Karakia.

Collaborations in this area of research

Dr. Louise Mainvil (Department of Human Nutrition) Dr. Margreet Vissers (Department of Pathology)

Potential funding bodies

University of Otago Research Grant was funded in October 2014

Location

Dunedin

Other relevant information

Relevance Score

Reference

_17913

Baseline and Follow up Survey Measures

Baseline Survey

Demographics [(age, gender, year at university, ethnic identify using NZ census categories, accommodation, and usual source of food/food preparation (usually eat residential food, cook for self, flatmates, etc.)]

The Big Five Inventory of Personality (44 items) (BFI; John & Srivastava, 1999)

*Mood Checklist (18 items) (Russell & Barrett, 1989)

*Fordyce Happiness Measure (1 item) (Fordyce, 1988)

*Flourishing Scale (8 items) (Diener, Wirtz et al., 2010)

*Self-rated Health and Vitality (5 items) (Short-Form 36; Ware & Sherbourne, 1992)

*Center for Epidemiologic Studies Depression Scale (20 items) (CES-D; Radloff, 1977)

*Anxiety subscale of the Hospital Anxiety and Depression Scale (7 items) (HADS; Zigmond & Snaith, 1983)

*Curiosity and Exploration Inventory-II (10 items) (CEI-II; Kashdan et al., 2009)

*Optimism measured by the Life Orientation Test –Revised, LOT-R (6 items) (Carver, Scheier, & Segerstrom, 2010)

*Creativity task - Divergent thinking (to come up with unusual uses of a standard brick given 3 minutes; Silvia et al., 2008)

Follow up Survey

* All measures starred above are repeated in the follow up survey

Self-reported supplement usage (multivitamins, vitamin C, vitamin B, vitamin D)

Self-reported current smoking status

Copies of full surveys available on request.

DAILY SURVEY

To be formatted to be accessed on a mobile phone. Participants will complete this survey once each day for 14 days. We will send the survey hyperlink to participants at 7 pm nightly using a commercial SMS system. Variable names are in bold.

Please answer these questions about your experiences today.

Circumplex Mood Items

Today, I felt...

		Not at all	*Slightly*	*Moderately*	*Very much*	*Extremely*
**DMOOD1**	happy	0	1	2	3	4
**DMOOD2**	tense	0	1	2	3	4
**DMOOD3**	angry	0	1	2	3	4
**DMOOD4**	enthusiastic	0	1	2	3	4
**DMOOD5**	relaxed	0	1	2	3	4
**DMOOD6**	sad	0	1	2	3	4

(Shortened) Daily Flourishing Scale (Diener, Wirtz et al., 2010)

		Strongly Disagree 1	2	3	Neither Agree nor Disagree 4	5	6	Strongly Agree 7
**DFS1**	Today, I led a purposeful and meaningful life.							
**DFS2**	Today, I was engaged and interested in my daily activities.							
**DFS3**	Today, I was a good person and lived a good life.							

Physical Health Measures-- Short-Form-36 (Ware & Sherbourne, 1992) Question 1 (DHEALTH) and Vitality Subscale (DVITAL1-4)

Overall, how would you say your health is today? **[DHEALTH]**

Poor	*Fair*	*Good*	*Very Good*	*Excellent*
0	1	2	3	4

	Today...	*None of the time 0*	*Some of the time 1*	*A little of the time*	*A good bit of the time*	*Most of the time 4*	*All of the time 5*

				2	3		
DVITAL1	Did you feel full of life?						
DVITAL2	Did you have a lot of energy?						
DVITAL3	Did you feel worn out?						
DVITAL4	Did you feel tired?						

Daily Physical Activity

How much time did you spend doing **vigorous and moderate** physical activities TODAY? _____ **minutes [DPAC]**

Daily Sun Exposure Questionnaire. Adapted from Glanz et al. (2008)

How many hours were you outside TODAY during daylight hours? **[DSUN] [Pull down menu]**

0	Less than 15 minutes
1	16 to 30 minutes
2	31 minutes to 1 hour
3	2 hours
4	3 hours
5	4 hours
6	5 hours
7	6 hours
8	7 hours
9	8 hours
10	9 hours
11	10 hours

Daily Curiosity Index (based on Curiosity and Exploration Inventory-II; CEI-II; Kashdan et al., 2009). Daily Creativity Index; concentration and motivation.

		Not at all 0	*Slightly 1*	*Moderately 2*	*Very much 3*	*Extremely 4*
**[DCURIOUS]**	How curious were you today? *(did you seek new things or experiences; look for opportunities to challenge yourself and grow as a person; or embrace the unfamiliar)*					
**[DCONCENT]**	Were you able to concentrate today? *(could you focus on studying or an activity)*					
**[DCREATE]**	How creative were you today? *(did you come up with novel or original ideas; express yourself in an original and useful way; or spend time doing artistic activities like, music, painting, writing, etc.)*					
**[DMOTIVATED]**	How motivated were you today? *(did you work towards your goals, or feel driven today)*					

INSTRUCTIONS: Now think about what you've eaten today, since waking up this morning. Remember to include all snacks and meals for the day including dinner. One serving size is roughly the amount you can fit in the palm of your hand.

How many **servings** of **vegetables** (fresh, frozen, or canned) did you eat? **Do not** include vegetable juices or hot chips. 1 serving = 1 large carrot **or** ½ cup cooked vegetables **or** 1 cup of salad
Pull down menu (none, <1 serving, 1 serving, 2 servings, 3 servings, 4 servings, 5 or more servings) **[DVEG]**

How many **servings** of **lollies, sweets, chocolate, or other confectionary items** did you eat TODAY? 1 serving = one regular sized chocolate bar
Pull down menu (none, <1 serving, 1 serving, 2 servings, 3 servings, 4 servings, 5 or more servings) **[DSWEETS]**

How many **servings** of **fruit** did you eat? **Do not** include fruit juice or dried fruit. 1 serving = 1 apple **or** 1 banana **or** 2 kiwifruits
Pull down menu (none, <1 serving, 1 serving, 2 servings, 3 servings, 4 servings, 5 or more servings) **[DFRUIT]**

How many **servings** of **hot chips, French fries, wedges, or kumara chips** did you eat? 1 serving = one cup **or** 1 small/regular fast food serving **or** ½ scoop of takeaway hot chips.
Pull down menu (none, <1 serving, 1 serving, 2 servings, 3 servings, 4 or more servings) **[DCHIPS]**

[use values of 0, .5, 1, 2, 3, 4, 5 for response options]

SUBMIT

After pressing SUBMIT - Thank you for completing today's survey!
